# Runx1 promotes scar deposition and inhibits myocardial proliferation and survival during zebrafish heart regeneration

**DOI:** 10.1242/dev.186569

**Published:** 2020-04-27

**Authors:** Jana Koth, Xiaonan Wang, Abigail C. Killen, William T. Stockdale, Helen G. Potts, Andrew Jefferson, Florian Bonkhofer, Paul R. Riley, Roger K. Patient, Berthold Göttgens, Mathilda T. M. Mommersteeg

**Affiliations:** 1 Department of Physiology, Anatomy and Genetics, University of Oxford, South Parks Road, Oxford OX1 3PT, UK; 2 MRC Molecular Haematology Unit, Weatherall Institute of Molecular Medicine, John Radcliffe Hospital, University of Oxford, Oxford OX3 9DS, UK; 3 Department of Haematology, Wellcome and MRC Cambridge Stem Cell Institute, University of Cambridge, Cambridge CB2 0AW, UK; 4 Micron Advanced Bioimaging Unit, Department of Biochemistry, South Parks Road, Oxford OX1 3QU, UK

**Keywords:** Heart, Regeneration, Runx1, ScRNA-seq, Zebrafish

## Abstract

Runx1 is a transcription factor that plays a key role in determining the proliferative and differential state of multiple cell types, during both development and adulthood. Here, we report how Runx1 is specifically upregulated at the injury site during zebrafish heart regeneration, and that absence of *runx1* results in increased myocardial survival and proliferation, and overall heart regeneration, accompanied by decreased fibrosis. Using single cell sequencing, we found that the wild-type injury site consists of Runx1-positive endocardial cells and thrombocytes that induce expression of smooth muscle and collagen genes. Both these populations cannot be identified in *runx1* mutant wounds that contain less collagen and fibrin. The reduction in fibrin in the mutant is further explained by reduced myofibroblast formation and upregulation of components of the fibrin degradation pathway, including plasminogen receptor annexin 2A as well as downregulation of plasminogen activator inhibitor *serpine1* in myocardium and endocardium, resulting in increased levels of plasminogen. Our findings suggest that Runx1 controls the regenerative response of multiple cardiac cell types and that targeting Runx1 is a novel therapeutic strategy for inducing endogenous heart repair.

## Abstract (precis)

**Highlighted Article:** The transcription-factor Runx1 orchestrates the injury response of many cardiac cell types, balancing collagen and fibrin deposition and clearance, as well as affecting myocyte proliferation and survival in zebrafish.

## INTRODUCTION

Heart regeneration potential varies considerably between species as well as with age. While zebrafish and neonatal mouse hearts can replace dead or lost cardiomyocytes rapidly with new heart muscle (Poss et al., 2002; Jopling et al., 2010; Porrello et al., 2011; Wang et al., 2011), medaka (Ito et al., 2014; Lai et al., 2017), cave fish (Stockdale et al., 2018), as well as adult mice and human hearts (Senyo et al., 2013), show only poor repair. Numerous studies are therefore looking into the underlying principles and mechanisms that promote, or prevent, effective cardiac regeneration to establish a basis for therapeutic intervention (González-Rosa et al., 2017). In models of successful regeneration, remaining cardiomyocytes have been shown to proliferate and to replace the fibrotic tissue with new heart muscle (Jopling et al., 2010; Kikuchi et al., 2010; Chablais et al., 2011; González-Rosa et al., 2011). This is dependent on a fine balance of interaction with other cell types, including the epicardium and endocardium (González-Rosa et al., 2017; Cao and Poss, 2018). Owing to the complexity of this interaction, we still lack a clear understanding of how the fibrotic tissue can be broken down and replaced by proliferating myocardial cells. Here, we report a role for Runx1 in regulating the delicate balance between collagen and fibrin degradation, and myocardial regeneration.

Runx transcription factors, which hetero-dimerise with core binding factor β (CBFβ), are transcription factors that can function as activators as well as repressors and, as such, are important regulators of lineage-specific cell fate. Runx1 [also known as acute myeloid leukaemia 1 protein (AML1) or core-binding factor subunit α 2 (CBFA2)] is a master transcription factor for determining the proliferative and differential state of multiple cell types, during both development and adulthood. Runx1 is most studied for its role in endothelial-to-haematopoietic transition during haematopoiesis in development (Lacaud et al., 2002; Chen et al., 2009; Lancrin et al., 2009; Boisset et al., 2010; Kissa and Herbomel, 2010; Lam et al., 2010) and as a well-known fusion oncogene (Silva et al., 2003; Blyth et al., 2005). Relatively little is known about the role of Runx1 in skeletal and heart muscle. It has been shown that Runx1 is important in skeletal muscle stem cell (SC) proliferation and its levels can affect the proliferative timing and thus the regenerative capacity of skeletal muscle cells (Umansky et al., 2015). In the heart, Runx1 is expressed in neonatal mouse cardiomyocytes and is upregulated in zebrafish, adult mouse, rat and human cardiomyocytes after injury (Gattenlöhner et al., 2003; Kubin et al., 2011; Eulalio et al., 2012; Górnikiewicz et al., 2016; Goldman et al., 2017). Conditional *Runx1* deficiency in mouse cardiomyocytes has been demonstrated to protect the mouse against the negative consequences of cardiac remodelling after myocardial infarction (McCarroll et al., 2018). Although no changes in injury size were found between myocardial conditional *Runx1* knockout and control mice, the remaining cardiomyocytes displayed improved calcium handling, accompanied by improved wall thickness and contractile function compared with wild type (McCarroll et al., 2018). However, as the knockout was cardiomyocyte specific, the involvement of other cardiac cell types was not investigated. In contrast to mouse, where constitutive Runx1 deletion is embryonically lethal, zebrafish *runx1^W84X^* mutants (Jin et al., 2012) are homozygote viable adults, allowing us to investigate the role of *runx1* loss of function during zebrafish heart repair down to the single cell level.

We show that Runx1 has important roles in the response of various cell types to injury, including thrombocytes, the epicardium, endocardium and myocardium. Thrombocytes are the fish equivalent of platelets and are important for blood clotting, with the difference that these are nucleated cells (Jagadeeswaran et al., 1999). We demonstrate that the removal of *runx1* leads to several unique cell type-specific responses within the heart, affecting cardiomyocyte proliferation and initial survival, deposition and degradation of fibrotic tissue/extracellular matrix at the wound site, and overall heart regeneration. The cellular composition of the wounded ventricle is altered between wild types and *runx1* mutants, with most noticeably a lack of thrombocytes and endocardial cells that express smooth muscle and collagen genes in the mutant. Additionally, the epicardium shows a reduction in the level of smooth muscle and collagen genes in the *runx1* mutant, on top of which there is a strong reduction in the number of cells clustering as myofibroblasts in *runx1* mutants. Additionally, there is a strong upregulation of components of the fibrin degradation pathway, including the annexin A2 complex. Taken together, our analysis suggests that heart regeneration is facilitated in the absence of *runx1* and identifies Runx1 inhibition as a potential therapeutic target to improve cardiac repair.

## RESULTS

### Runx1 becomes widely expressed in zebrafish hearts after injury

**Fig. 1.**
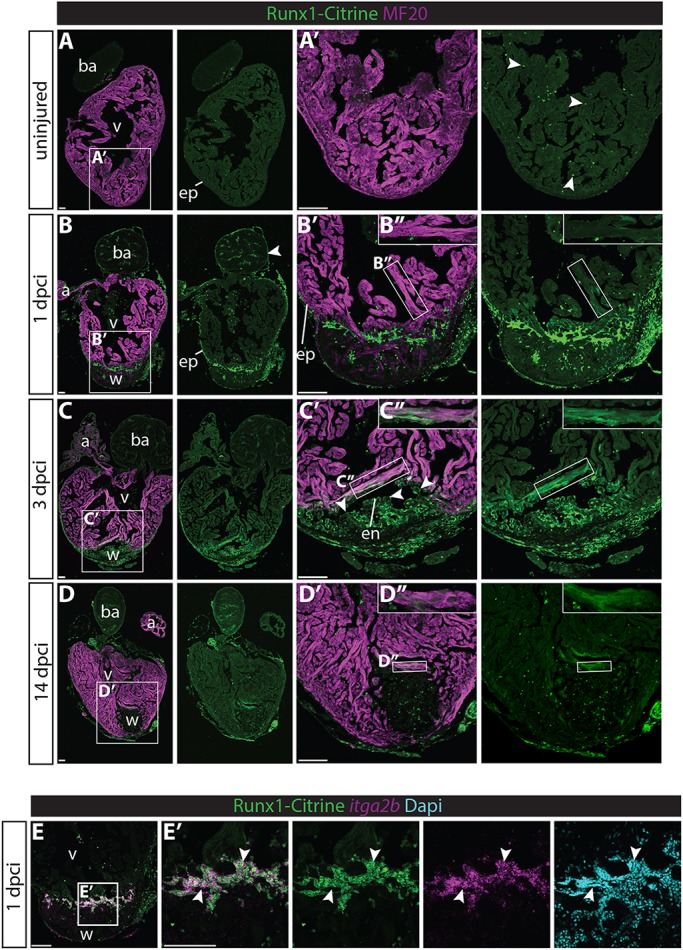
**Runx1-Citrine becomes strongly expressed in the heart after cryo-injury.** (A-D″) Immunohistochemistry for Runx1-Citrine (GFP antibody) and the myocardial marker MF20 at different time points after cryo-injury. (A,A′) Citrine expression in the uninjured hearts was confined to a small number of cells scattered around the heart (arrowheads). (B-B″) At 1 dpci, the epicardium was Citrine positive (arrowheads). Bright blood cells were visible within the wound and there was dim expression of Citrine overlapping with MF20 (B″). (C-C″) At 3 dpci, the epicardium, endocardium (arrowheads) and other wound cells were positive for Citrine. In addition, the myocardium in the border zone next to the wound was highly Citrine positive (C″). (D-D″) Expression of Citrine diminishes at 14 dpci but is still visible, especially in the myocardium (D″). (E,E′) *In situ* hybridisation for *itga2b* with immunohistochemistry for Runx1-Citrine and nuclear marker DAPI. Arrowheads indicate overlap of Runx1-Citrine with *itg2b* mRNA, indicating that thrombocytes are positive for Runx1-Citrine. a, atrium; ba, bulbus arteriosus; dpci, days post cryo-injury; en, endocardium; ep, epicardium; v, ventricle; w, wound. Scale bars: 100 μm.

To evaluate *runx1* expression in the adult heart, we induced cryo-injury using a liquid nitrogen-cooled probe in the *Tg(BAC-runx1P2:Citrine)* zebrafish line, in which cytoplasmic Citrine fluorescence is placed under the control of the *runx1* P2 promoter (Bonkhofer et al., 2019). Although several other transgenic *runx1* reporter zebrafish lines have been published, these are either enhancer lines (Goldman et al., 2017) or the *Tg(runx1P2:EGFP)* line with a short promoter sequence displaying ectopic expression during development (Lam et al., 2009, 2010). This prompted us to use a line with a larger regulatory region (Bonkhofer et al., 2019). The P2 promoter is the main one of two *runx1* promoter regions known to drive expression in definitive hematopoietic stem cells (HSCs) in the dorsal aorta during development (Lam et al., 2010); however, its expression in the adult heart is unknown. In the uninjured heart, Runx1-Citrine expression was sparse but present in a small number of cells spread throughout the heart, mostly blood cells (Fig. 1A,A′). However, after injury, expression became much more widespread: 1 day post cryo-injury (dpci), a large collection of bright Citrine-positive cells was present in the injury site (Fig. 1B,B′), indicating the presence of Citrine-positive blood cells in the wound. In addition to the blood cells, other cell populations started to express Citrine, including cells within the epicardium all around the heart (arrowheads, Fig. 1B). Additionally, weak expression of Citrine was observed in cardiomyocytes bordering the injury site (Fig. 1B′,B″, insert). Three days after injury, Citrine expression in these cell types was even more pronounced, especially within the endocardium specifically near the injury site (arrowheads, Fig. 1C-C″). Moreover, at this time-point, myocardial cells surrounding the injury site strongly expressed Citrine, as shown by overlapping expression of Citrine with the myocardial marker MF20 (Fig. 1C-C″, insert). This pattern was maintained at 7 dpci, but started to taper-off around 14 dpci (Fig. S1A-A″, Fig. 1C-D″). Even in sham-operated hearts, in which the ventricle was only exposed to a room temperature probe, Citrine expression was upregulated in both the epicardium (arrowheads) and myocardium, but not in the endocardium (Fig. S1B-B″). To verify the cell type-specific expression of Citrine, we confirmed overlapping expression with different cell-type specific markers. The bright blood cell population present in the wound at 1 dpci was also highly positive for *itga2b*, which is a marker for nucleated thrombocytes (arrowheads, Fig. 1E,E′) (Albert and Christopher, 2013). Additionally, we found Citrine overlapping with leukocyte marker LyC, endothelial/endocardial marker ERG1 and epicardial/fibroblast marker *tcf21* (Fig. S1C-E) at 3 dpci. As *runx1* expression was analysed by visualisation of a transgene, we also checked whether transgene expression followed the same pattern as endogenous runx1 RNA using RNA-scope *in situ* hybridisation (Wang et al., 2012). Runx1 RNA and Citrine expression showed clear overlapping expression patterns, with RNA present in the Citrine-positive epicardium, myocardium and endocardium (Fig. S2A-C) after injury. Runx1 RNA was absent or expressed at very low levels in the Citrine-negative myocardium of the rest of the ventricle (Fig. S2B,C, asterisks). To summarise, *runx1* expression becomes strongly upregulated in several cell populations of the heart after injury, in the myocardium, endocardium and epicardium surrounding the wound area. This upregulation of Runx1-Citrine after cryo-injury suggests a role for *runx1* in multiple cell types during heart regeneration.

### Faster compact wall regeneration in *runx1* mutants compared with wild-type zebrafish

**Fig. 2.**
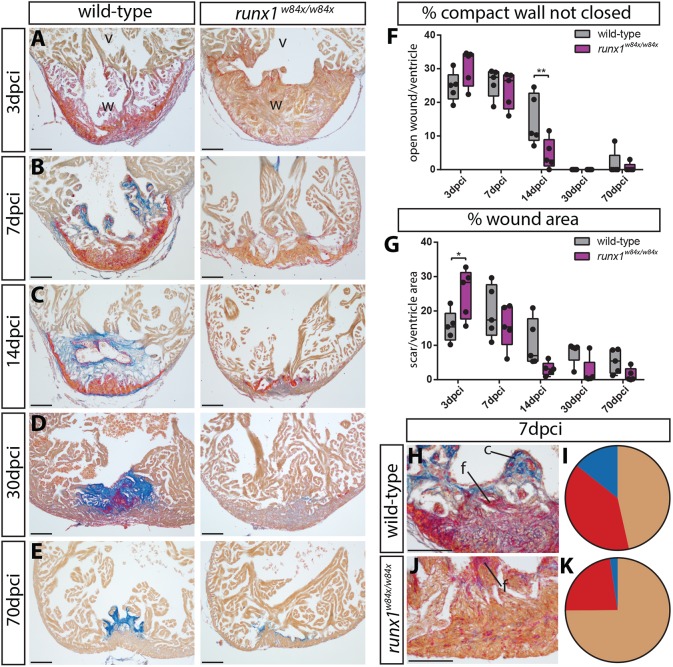
**Different wound composition and faster regeneration in *runx1* mutant compared with wild-type hearts.** (A-E) AFOG staining of wild-type and *runx1* mutant ventricles at five different time points after injury. (F,G) Quantification of the difference in wound size between the wild type and mutant at the different time points, measured by the percentage of the compact wall not yet closed (F) and the percentage of wound area compared with total ventricle area (G). *n*=5 per time point, two-way ANOVA with Sidak test. *P<0.05 and **P<0.01. Box extends from the 25th to 75th percentiles and whiskers indicate minimum to maximum with all data points shown. (H-K) Quantification of differences in wound composition between the fish at 7 dpci (collagen, blue; fibrin, bright red; all other cells, including myocardium/blood cells are orange), *n*=5. c, collagen; f, fibrin; v, ventricle; w, wound. Scale bars: 100 μm.

Based on the observed expression of Runx1 after injury, we questioned whether heart regeneration is affected in absence of *runx1*? We used the adult homozygote viable global *runx1^W84X/W84X^* mutant, which has a premature STOP codon truncation mutation, leading to an almost complete loss of the Runt domain and loss of function (Jin et al., 2009; Sood et al., 2010). After performing cryo-injury on *runx1* wild-type and mutant fish, we isolated the hearts at five different time-points, from 3 to 70 days post injury. Acid Fuchsin Orange G staining (AFOG, labelling collagen in blue, fibrin in bright red and all other cells, including the myocardium/blood cells, in orange) showed a clear difference between wild-type and *runx1* mutant hearts (Fig. 2A-K). At 3 dpci, the injury site was clearly visible in both fish, but the wild-type hearts showed a much more extensive deposition of bright red fibrin compared with the mutants (Fig. 2A). As well as less fibrin deposition, we also observed reduced collagen (blue) deposition at 7 dpci in the mutants (Fig. 2B,H-K). Whereas the wild-type wound consisted on average of 39.2% fibrin (red) blood clot and 14.3% collagen (blue), *runx1* mutant hearts had around 22.7% fibrin (red) and 2.4% collagen (blue) labelling. Despite these differences in wound composition, comparison of the wound size did not show any significant differences between the wild-type and mutant hearts at 3 and 7 dpci (Fig. 2A,B,F,G). However, at 14 dpci, there was a significantly stronger decrease in open wound length in the mutant compared with the controls, indicating a faster resolution of the lesion (Fig. 2C,F,G). At 30 dpci both mutants and controls had closed the compact myocardial wall over the wound, indicating good overall repair progress, but the remaining internal lesion was less pronounced in the mutants, with blue collagen mainly present in between the regenerated trabeculae (Fig. 2D). The difference was still visible at 70 dpci, with trace amounts of blue collagen label present in the mutants. These data show that the *runx1* mutants have a significantly larger area of their compact wall closed at 14 dpci compared with wild types and deposit a different fibrotic tissue/extracellular matrix after heart injury compared with wild types.

### Increased myocardial proliferation and myocardial protection against cryo-injury in the *runx1* mutant

**Fig. 3.**
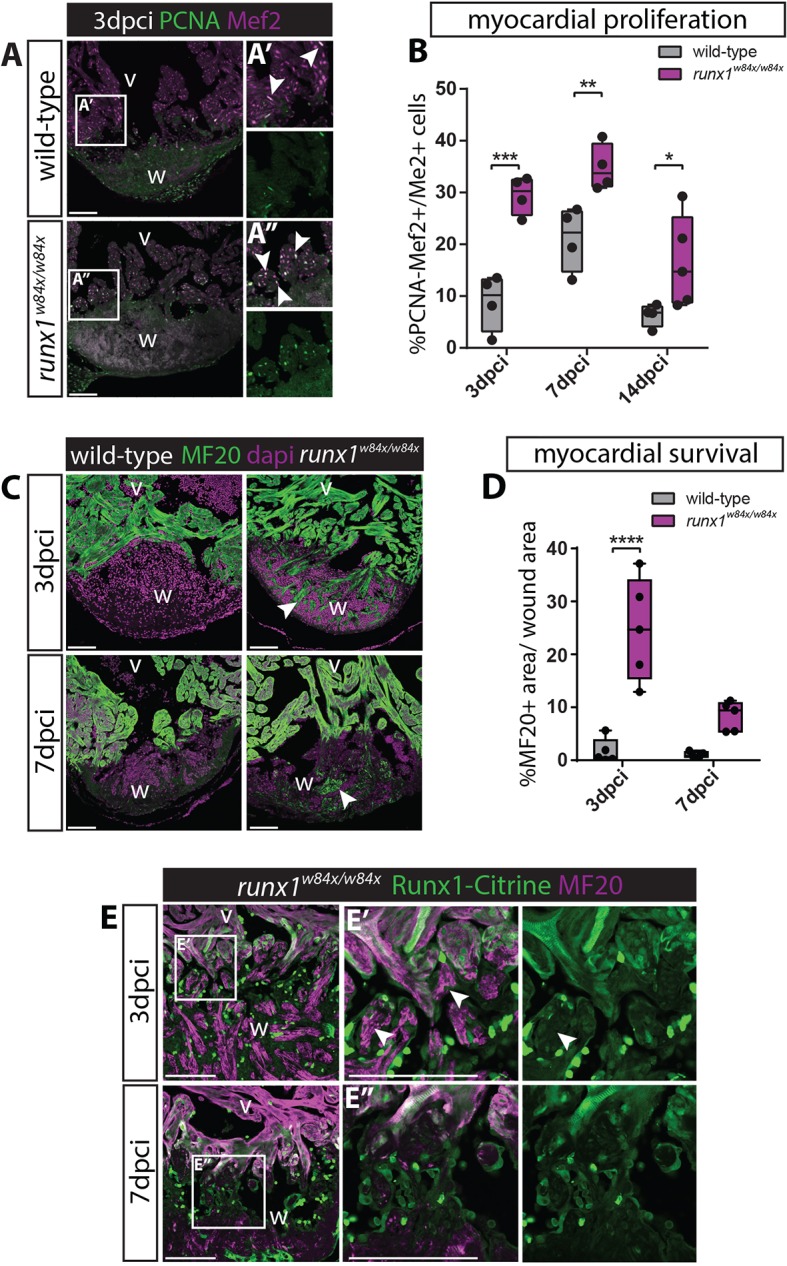
**Increased myocardial proliferation and protection in the *runx1* mutant.** (A-A″) Immunohistochemistry for PCNA and Mef2 on 3 dpci sections. An increased number of double-positive cells (arrowheads) seems present in the mutant compared with the wild-type wound border. (B) Quantification of PCNA-positive proliferating Mef2-positive myocardial cells after injury shows increased myocardial proliferation in the *runx1* mutant at all time-points analysed. *n*≥4, two-way ANOVA with Sidak test. (C) Immunohistochemistry for MF20 with the nuclear marker Dapi. Arrowheads indicate the presence of MF20-positive myocardial cells in the wound in the mutant at both 3 and 7 dpci. (D) Quantification of the MF20-positive area in the wound on sections in the wild type and mutant shows increased presence of myocardial cells in the mutant. *n*=5, two-way ANOVA with Sidak test. *P<0.05, **P<0.01, ***P<0.001 and ****P<0.0001. Box extends from the 25th to 75th percentiles and whiskers indicate minimum to maximum with all data points shown. (E) Immunohistochemistry for Citrine and MF20. Arrowheads indicate the surviving MF20-positive cells in the mutant wound that are Runx1-Citrine negative. v, ventricle; w, wound. Scale bars: 100 µm.

As we found that Runx1-Citrine expression was activated in cardiomyocytes that were in direct contact with the wound (Fig. 1), and these border-zone cardiomyocytes are known to be highly proliferative and to contribute new cardiomyocytes to the wound (Jopling et al., 2010), we wanted to investigate whether the more rapidly healing *runx1* mutants show increased myocardial proliferation. To test this, proliferating cells were labelled on sections with proliferating cell nuclear antigen (PCNA) and myocardial nuclei with Mef2. Myocardial proliferation was significantly increased near the wound in mutants compared with the wild types at all time-points analysed (Fig. 3B). Thus, Runx1 expression in the wound border zone cardiomyocytes appears to inhibit myocardial proliferation. Additionally, in the *runx1* mutant wound, we also observed a large number of myocardial cells surviving after cryo-injury, most notably at 3 dpci, that were absent in wild-type injured hearts and independent of initial wound size (Fig. 3C,D). These surviving myocardial cells had strongly reduced in number by 7 dpci in the mutant, suggesting that this protection is likely temporary and that these cells do not survive long term (Fig. 3C,D). They did not express Runx1-Citrine and had lost their normal myofibril/sarcomere structure (Fig. 3E). This initial protection of cardiomyocytes against injury resembles the cardio-protective effect described in mice, which was found to be linked to an improved calcium uptake of the sarcoplasmic reticulum (McCarroll et al., 2018). To establish whether the increase in myocardial proliferation and survival are linked, we also performed resection injury during which a small region of the ventricular apex was removed using fine scissors, excluding any myocardial survival within the wound. After resection injury, both myocardial proliferation and compact wall regeneration were not significantly different between the mutants and wild types (Fig. S3A-D), indeed suggesting that there could be a link, even though intuitively better survival implies less demand for proliferation. Taken together, these findings reveal pleiotropic roles for Runx1 in reducing cardiomyocyte proliferation and survival following heart injury.

### Significant increase in Runx1-Citrine-positive endocardial cells after injury

**Fig. 4.**
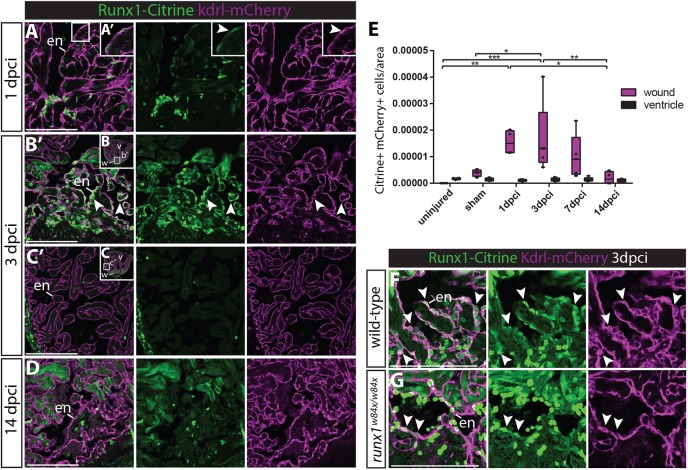
**Runx1-Citrine positive endocardial cells appear in the wound after injury.** (A-D,F,G) Immunohistochemistry analysis for Citrine- and mCherry-positive cells in the *Tg(BAC-runx1P2:Citrine;kdrl:Hsa.HRAS-mCherry)* line. (A,A′) The wound at 1 dpci, with the box highlighting the flat and weakly Citrine- and mCherry-positive endocardial cells in the wound (arrowhead). (B-C′) Clearly visible and round Citrine- and mCherry-positive cells in the wound at 3 dpci (B,B′, arrowheads), but not further away from the wound (C,C′). (D) At 14 dpci, not many double-positive cells are visible anymore. (E) Quantification of the number of Citrine and mCherry double-positive cells in and away from the wound, *n*=4, two-way ANOVA with Tukey's test. *P<0.05, **P<0.01 and ***P<0.001. Box extends from the 25th to 75th percentiles and whiskers indicate minimum to maximum with all data points shown. (F,G) *runx1* mutant wounds have a reduced number of double-positive cells (arrowheads). en, endocardium; v, ventricle; w, wound. Scale bars: 100 μm.

As we also observed strong expression of Runx1-Citrine in the endocardium, which plays crucial roles during regeneration (González-Rosa et al., 2017; Münch et al., 2017; Sánchez-Iranzo et al., 2018), we next analysed the *runx1*-expressing endocardium in more detail (Fig. 4A-F). We crossed the *Tg(BAC-runx1P2:Citrine)* with the *Tg(kdrl:Hsa.HRAS-mCherry)* line (Chi et al., 2008), which in combination label Runx1-Citrine endothelial/endocardial cells with membrane mCherry fluorescence, and initially observed very few Citrine-mCherry double-positive cells in intact unopened or sham-operated hearts (Fig. 4E). At 1 dpci, we observed a significant increase in mCherry-positive cells in the wound area with a flat endocardial cell morphology and dim Citrine expression compared with the bright Citrine-positive blood cells (Fig. 4A′,E). Double-positive cells were most clearly visible at 3 dpci (Fig. 4B,B′,E), with a rounder cell morphology, while throughout the remaining intact ventricle away from the wound, only few cells were observed at all stages analysed (ventricle, Fig. 4C,C′,E). This Citrine/mCherry double positive population was still highly present at 7 dpci, but decreased towards baseline levels at 14 dpci (Fig. 4D,E). The known functions of Runx1 (Lacaud et al., 2002; Chen et al., 2009; Lancrin et al., 2009; Boisset et al., 2010; Kissa and Herbomel, 2010; Lam et al., 2010), combined with its extensive expression pattern in the endocardium, suggests a novel role for *runx1* in the endocardium during regeneration.

### Single cell sequencing identifies subpopulations of *runx1*-expressing cells after injury

**Fig. 5.**
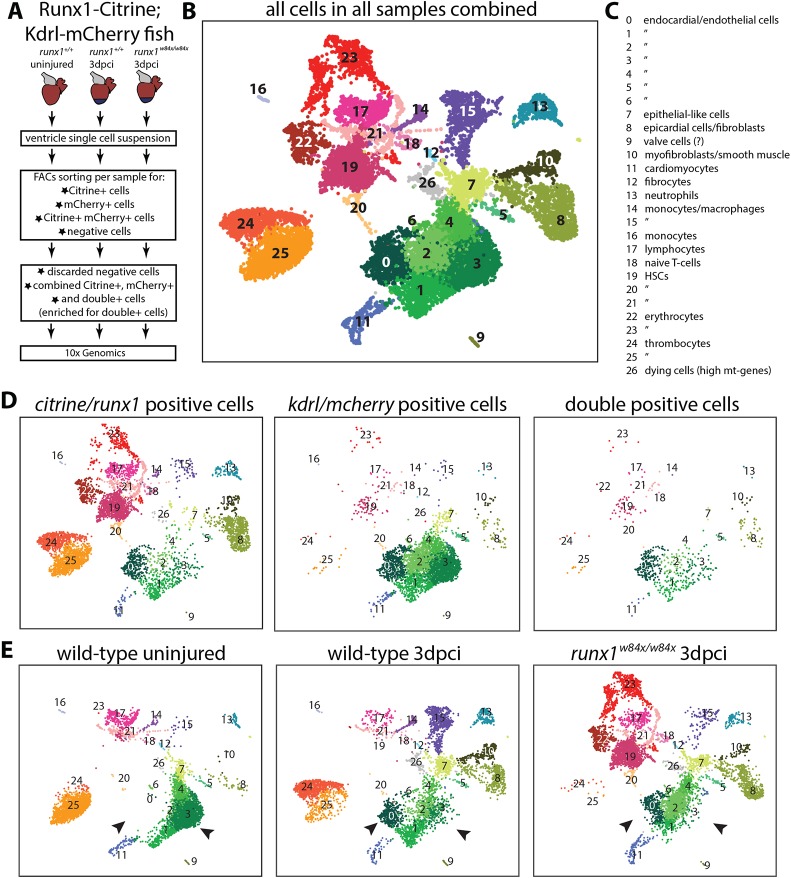
**Single cell sequencing of Citrine- and mCherry-positive cells.** (A) Experimental design of selection of cells for single cell sequencing using the 10x Genomics platform. (B) UMAP plot of all cells combined, clustering into 27 different clusters. (C) Annotation of the different cell clusters. (D) UMAP plot separated into *citrine/runx1*-positive cells, *mcherry/kdrl*-positive cells and double-positive cells. (E) UMAP plot separated into wild-type uninjured cells, wild-type 3 dpci cells and *runx1* mutant 3 dpci cells. Arrowheads indicate the shift in endocardial/endothelial cells, with C0 and C2 appearing, and C3 reducing in size after injury. HSC, haematopoietic stem cells; mt, mitochondrial.

To further investigate the function of Runx1 in all *runx1*-expressing cell types after injury, with a focus on the endocardium/endothelium, we performed single cell sequencing using the 10x Genomics platform. The *runx1^W84X/W84X^* mutant line was crossed into the *Tg(BAC-runx1P2:Citrine;kdrl:Hsa.HRAS-mCherry)* background and confirmed for preserved BAC-Runx1P2:Citrine expression (Fig. S4A). Citrine expression was overall similar to that in the wild-type heart, including expression in endocardial cells. However, we observed a reduction in the number of Citrine and mCherry double-positive cells in the mutant wound (Fig. 4F,G). The ventricles of *runx1* wild-type uninjured, *runx1* wild-type 3 dpci and *runx1* mutant 3 dpci *Tg(BAC-runx1P2:Citrine;kdrl:Hsa.HRAS-mCherry)* fish were dissociated and FACS sorted (Fig. 5A). By FACS sorting for Citrine and mCherry, we found a 4.5-fold increase in the number of double-positive cells in wild-type 3 dpci hearts compared with wild-type uninjured hearts, confirming our image-based cell counts (Fig. S4B, Fig. 4E). Mutant hearts had only one-third of the amount of double-positive cells after injury compared with wild types (Fig. S4B). Before sequencing, we excluded negative cells and combined all single-positive and double-positive cells, while enriching for the double-positive population that might otherwise have been missed during single cell analysis, owing to their low numbers. Sequencing and subsequent clustering of all cells combined led to the identification of 27 cell clusters (C) that comprised all the expected cell populations, including endocardial/endothelial cells, myocardial cells, epicardial cells, myofibroblasts, thrombocytes and different leukocyte populations (Fig. 5B,C, Fig. S5A). Kdrl/mCherry mRNA-positive cells were mainly present in a large group of closely related cell clusters (C0-6), whereas runx1/Citrine mRNA-positive cells were, as expected, present in all clusters; double-positive cells largely grouped in the main kdrl/mCherry group (Fig. 5D). In the uninjured heart, endocardial/endothelial cells grouped into three main distinct clusters (C1, C3 and C4), indicating a degree of heterogeneity within these cell populations (Fig. 5E). After injury in both wild-type and *runx1* mutant hearts, two large additional endocardial/endothelial cell populations appeared (C0 and C2), while C3 was smaller (arrowheads, Fig. 5E). These injury-specific endocardial populations were highly positive for *serpine1* expression (Fig. S5B), indicating that this population is largely similar to the previously identified highly mobile *serpine1*-positive endocardial population (Münch et al., 2017), which was confirmed on sections (Fig. S5C,C′, arrowheads). Although Citrine-positive populations of neutrophils and macrophages were present in both the mutant and wild type after injury (C13 and C15), we observed differences in other blood cell populations and, most notably, mutant hearts lacked an obvious cluster of mature thrombocytes and monocytes (C24-25 and C16, Fig. 5B-E). In contrast, other blood cell clusters unique to the mutant were present (C19, C22 and C23), and characterised by highly expressed genes such as *gata2b* or *myb* (Fig. S5D). Analysis of wild-type and mutant tissue sections confirmed the unique presence of these abnormal blood cell populations in the mutant, resulting in an altered leukocyte profile in the wound after injury (Fig. S5E-H′). Although nonsense-medicated decay (El-brolosy et al., 2019) could be a possibility in the *runx1* mutant, we did not find upregulation of other compensating Runx genes (*runx2a*, *runx2b* and *runx3*). These results show, on a single cell basis, how Runx1 becomes activated after injury with specific cell composition differences between the mutant and wild-type hearts.

### Runx1-positive endocardial cells express smooth muscle genes

**Fig. 6.**
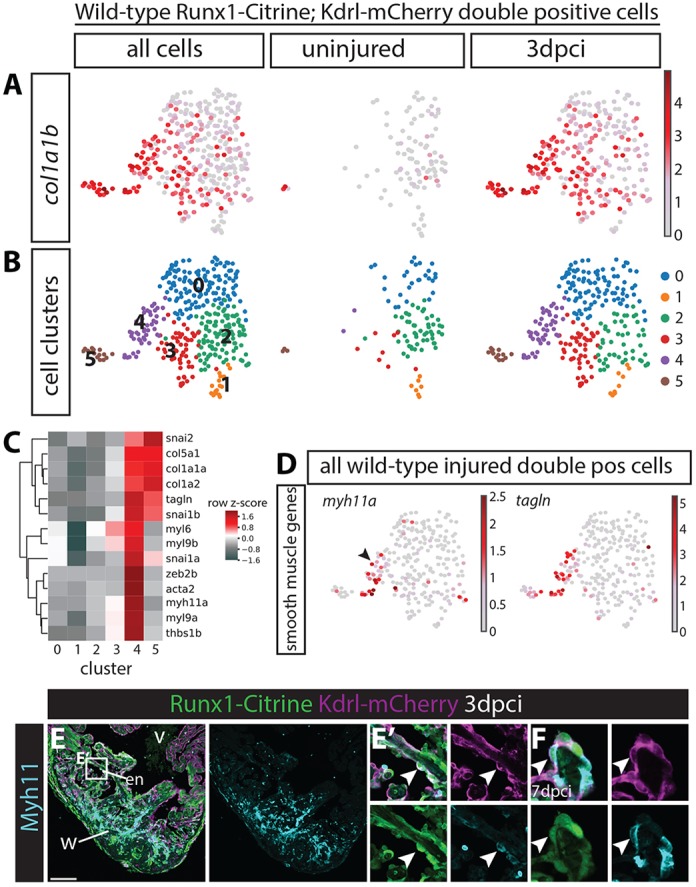
**Subset of wild-type double-positive cells expresses smooth muscle genes.** (A,B) All wild-type uninjured and 3 dpci *Tg(BAC-runx1P2:Citrine;kdrl:Hsa.HRASmCherry)* double-positive cells visualised in an UMAP plot. (A) Expression of *collagen 1a1b* specifically in 3 dpci double-positive cells. (B) Cell clustering within the double-positive population identifies six different cell clusters. (C) Heatmap showing that both clusters 4 and 5 express high levels of collagens, whereas cluster 4 specifically expresses smooth muscle genes. (D) *myh11a* and *tagln* are expressed in cluster 4 after injury. Arrowhead indicates *myh11a* expression in cell cluster 4. (E-F) Immunohistochemistry for Citrine, mCherry and Myh11. Arrowheads indicate expression of Myh11 in *Tg(BAC-runx1P2:Citrine;kdrl:Hsa.HRAS-mCherry)* double-positive cells at 3 (E,E′) and 7 (F) dpci. en, endocardium; v, ventricle; w, wound. Scale bars: 100 μm.

Within the individual *runx1-Citrine*-expressing cell groups, we focussed next on the endocardial/endothelial cells in the wild-type uninjured and wild-type 3 dpci hearts. Citrine/mCherry double-positive endocardial cells showed a highly injury-specific upregulation of collagens, e.g. *collagen 1a1b* (Fig. 6A). Upregulation of collagens in the wound endocardium has been observed before (Münch et al., 2017; Sánchez-Iranzo et al., 2018), but has not previously been analysed using single cell transcriptomics. Clustering of the Citrine/mCherry double-positive cells showed two clusters (C4 and C5) appearing after injury, with upregulation of genes involved in extracellular matrix formation as well as endothelial-to-mesenchymal transformation (EMT, *snai1a/b*, *snai2* and *zeb2b*) (Fig. 6B,C, Fig. S6A). Interestingly, the endocardial cells of cluster 4 can be identified by specifically upregulated smooth muscle genes, with high expression of *myh11a*, *myl6* and *myl9a/b*. *tagln* (*sm22a*) was expressed in both clusters 4 and 5, but strongest in cluster 4 (Fig. 6C,D). Although smooth muscle gene expression has been suggested previously in the border zone endocardium (Wu et al., 2016), we were able to confirm the expression of smooth muscle gene *myh11* in endocardial cells on sections (arrowheads in Fig. 6E,F, Fig. S6B). These data combined suggest that the *runx1*-positive endocardium is a heterogeneous cell population, with subsets of cells starting to express collagens, EMT genes and/or smooth muscle genes after injury. Although the expression of EMT genes suggests that these endocardial cells could become mesenchymal, recent lineage tracing of the endocardium did not point to contribution of the endocardium to collagen-forming cells (Sánchez-Iranzo et al., 2018). As the combination of high collagen and smooth muscle gene expression is considered a hallmark of myofibroblasts, we investigated this in more detail and expanded our analysis to the *runx1* mutants.

### Smooth muscle genes are expressed in the endocardium and thrombocytes, but both these cell clusters are absent in the *runx1* mutant

**Fig. 7.**
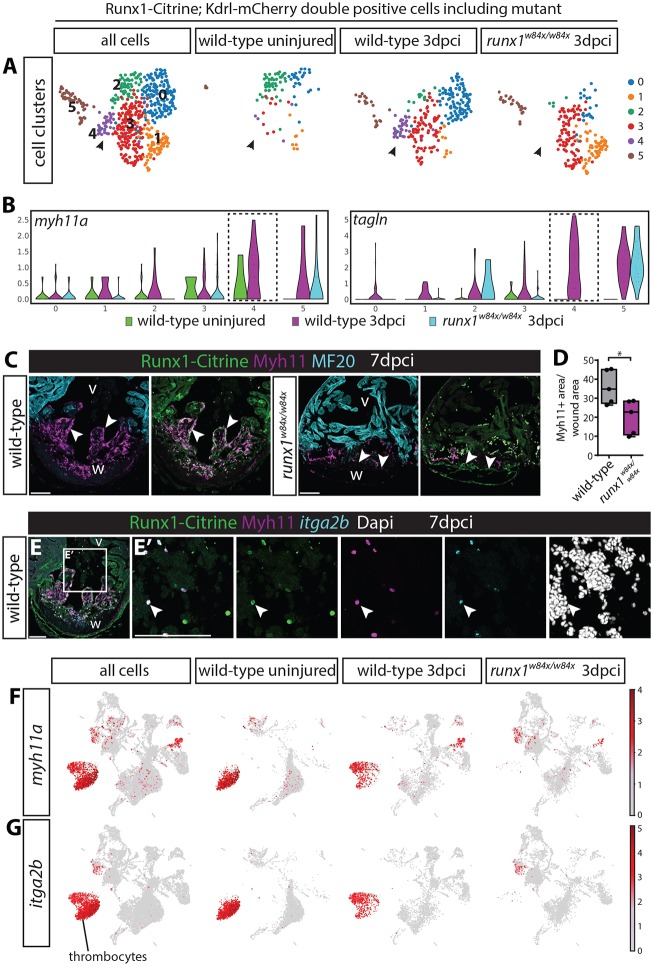
**Endocardial and thrombocyte Myh11-positive populations are strongly reduced in the *runx1* mutant.** (A) UMAP plot of *Tg(BAC-runx1P2:Citrine;kdrl:Hsa.HRASmCherry)* double-positive cells in the 3 dpci *runx1* mutant as well as uninjured and 3 dpci wild-type cells. Arrowheads indicate the double-positive cluster 4 that is absent in the *runx1* mutant. (B) Violin plot showing that the absent cluster 4 is the cluster most strongly expressing smooth muscle genes in the wild type after injury. (C) Immunohistochemistry for Citrine, Myh11 and MF20, showing reduced staining for Myh11 in the *runx1* mutant wound at 7 dpci compared with the wild type. Arrowheads indicate overlap of Myh11 with Citrine in the endocardium. (D) Quantification of the area of Myh11 expression in the wound on sections between mutants and wild types. *n*=5, unpaired two-tailed *t*-test. *P<0.05. Box extends from the 25th to 75th percentiles and whiskers indicate minimum to maximum with all data points shown. (E,E′) *In situ* hybridisation for *itga2b* combined with immunohistochemistry for Citrine and Myh11 with nuclear marker Dapi. Arrowheads indicate Myh11-expressing blood cells that express the thrombocyte marker *itga2b*. (F,G) UMAP plot of all cells confirms expression of *myh11a* in the *itga2b*-positive thrombocyte cluster. v, ventricle; w, wound. Scale bars: 100 μm.

**Fig. 8.**
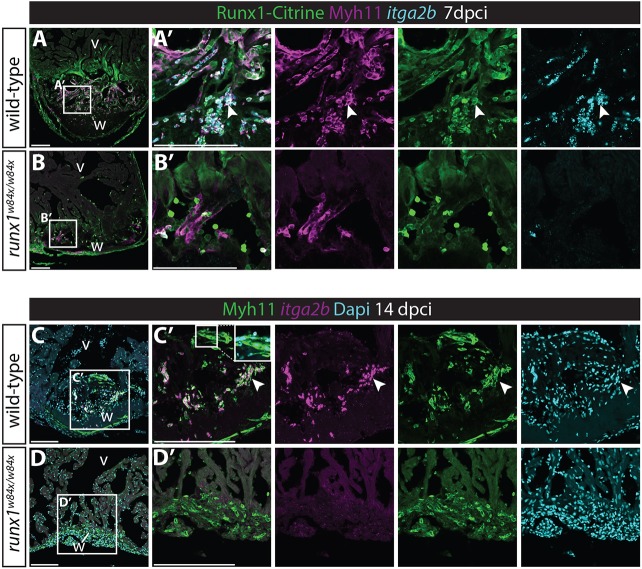
**Myh11-positive endocardial cells and thrombocytes retain their double identity.** (A-B′) *In situ* hybridisation for *itga2b* combined with immunohistochemistry for Citrine and Myh11. Arrowheads indicate Myh11-positive *itga2b*-positive thrombocytes present in the wild-type wound that are largely missing in the *runx1* mutant wound at 7 dpci. (C-D′) *In situ* hybridisation for *itga2b* combined with immunohistochemistry for Myh11 with nuclear Dapi staining. Both the endocardium (inset) and thrombocytes (arrowheads) still express Myh11 in the wild-type wound at 14 dpci, while being absent in the mutant wound. v, ventricle; w, wound. Scale bars: 100 μm.

We first wanted to see whether the lower number of Citrine/mCherry double-positive cells in the mutant produced a similar smooth muscle profile to the injured wild type, so we included the *runx1* mutant in our analysis of double-positive cells from the single cell data. Although cluster 5 cells were present, the smooth muscle gene expressing endocardial cluster 4 was completely absent in the *runx1* mutant (arrowheads, Fig. 7A,B), as were the cells most strongly expressing *myh11a* and *tagln* (Fig. 7B). Analysis of Myh11 expression in the wound confirmed that much fewer Myh11-positive cells were found in the mutant heart sections; however, the strongly reduced area of cells expressing Myh11 in the mutant (Fig. 7C,D) was too large to be attributed exclusively to endocardial cells. While analysing endocardial Myh11 presence via antibody staining, we also noticed Myh11 presence in circulating blood cells in wild-type hearts (arrowheads, Fig. 7E,E′). Myh11 is often considered to be marker for smooth muscle and myofibroblasts, but in addition to being present in endocardial cells, Myh11 staining also clearly overlapped with strong *itga2b*-expressing cells, identifying these cells as thrombocytes (Fig. 7E,E′). The single cell data in turn confirmed that the *itga2b*-positive thrombocyte populations highly express *myh11a* (Figs 5B,C and 7F,G) and we confirmed the accumulation of Myh11/*itga2b*-positive thrombocytes in the wound area after injury (arrowheads, Fig. 8A). Also in line with the single cell data, we found that the Myh11-positive thrombocyte population was largely absent in the mutant (Fig. 8A-B′). This means that both smooth muscle gene-expressing cell types, the Myh11-positive endocardium and thrombocytes, are behaving differently in the mutant. It raises the questions of what happens to these cells over time in the wild-type situation and how that relates to the observed differences in fibrin and collagen between the wild types and mutants in the AFOG stained sections (Fig. 2)? At 14 dpci, we found that Myh11 is still largely present in the endocardium and thrombocytes in wild types (arrowheads, Fig. 8C-D′). This double identity could mean that part of the fibrosis-forming cells in fish do not completely differentiate into full myofibroblasts, but remain endocardial or thrombocyte in nature. We cannot exclude the possibility that at least some cells lose the expression of the endothelial and thrombocyte markers; however, at 14 dpci, when the wound size is already reducing, a large number of Myh11-positive cells on the luminal side of the wound still have a thrombocyte or endocardial profile. Interestingly, most collagen (blue) deposition in the wound seen in the AFOG staining (Fig. 2B,C) was observed near the location of the Myh11-expressing endocardial cells and thrombocytes, and to a much lesser extent near the epicardium (Figs 2B,C and 8A). The strong reduction of both the blue AFOG staining and Myh11-positive cells in the mutant suggests, therefore, that a large proportion of this collagen could be deposited by the Myh11-positive endocardial cells and thrombocytes, instead of myofibroblasts. A reduction of these cells could, therefore, potentially explain the different wound composition, as well as the improved resolution of fibrotic tissue during heart regeneration in the *runx1* mutant. This is a novel and surprising finding, as the epicardium is considered to be the main source of myofibroblasts and collagen deposition in the heart (Kikuchi et al., 2011; González-Rosa et al., 2012; Sánchez-Iranzo et al., 2018).

### Changes in Runx1-Citrine positive epicardial cells after injury

**Fig. 9.**
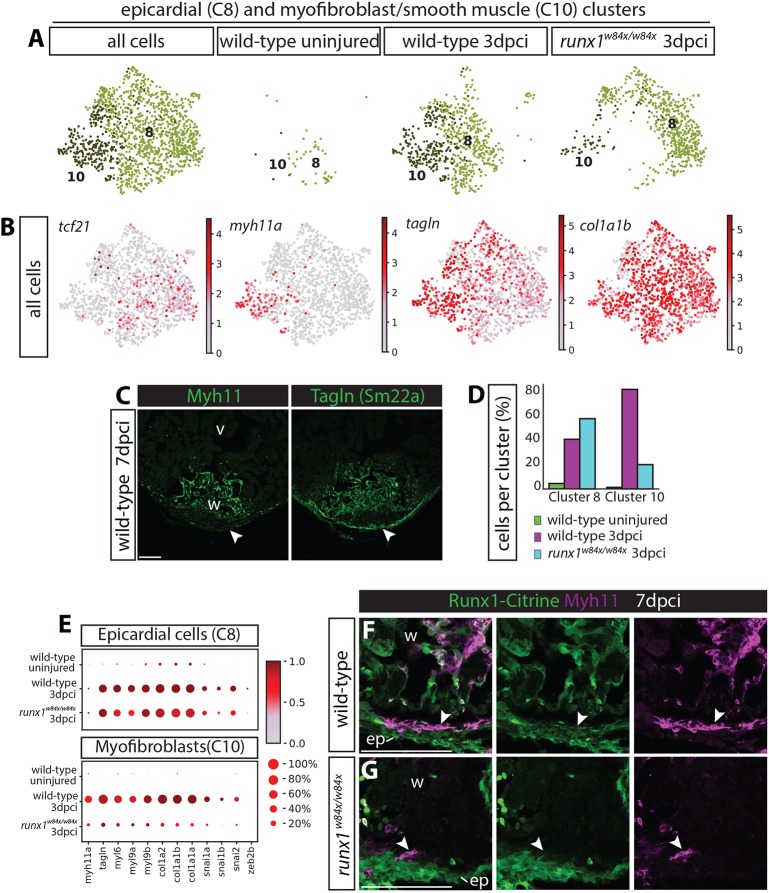
**Reduction in myofibroblast numbers in the *runx1* mutant.** (A) UMAP plot combining cluster 8 and 10 from Fig. 4B, showing very few cells in these clusters in the uninjured wild type, but the appearance of both populations after injury in the wild type and *runx1* mutant. (B) UMAP plot from A, indicating expression levels of *tcf21*, *myh11a*, *tagln* and *col1a1b*. The epicardial cluster 8 expresses *tcf21*, whereas myofibroblast cluster 10 expresses *myh11a*. Both clusters express *tagln* and *col1a1b*. (C) Staining of 7 dpci sections confirms presence of Tagln and absence of Myh11 in the epicardium (arrowheads). (D) Single cell data showing the numbers of cells per cluster per sample. Increased number of epicardial cells and a reduced number of myofibroblast cells in the *runx1* mutant compared with the wild type. (E) Dotplot showing expression levels of smooth muscle, EMT and collagen genes per sample in clusters 8 and 10. Increased cell numbers and expression of myofibroblast genes in both the epicardial and myofibroblast clusters in the injured wild-types compared to the uninjured wild-types. Epicardial myofibroblast gene expression is lower in the *runx1* mutant compared to the uninjured wild-type, while the number of myofibroblast cells is strongly reduced in the mutant. (F,G) Immunohistochemistry for Citrine and Myh11 on 7 dpci sections. Analysis of Myh11 on 7 dpci sections confirmed the reduction in myofibroblast cell numbers close to the epicardium (arrowheads). ep, epicardium; v, ventricle; w, wound. Scale bars: 100 μm.

As the epicardium has been shown to contribute myofibroblasts to the wound in zebrafish (González-Rosa et al., 2012; Sánchez-Iranzo et al., 2018), and we observed activation of Runx1-Citrine in the epicardium, we next compared the epicardial cluster to the myofibroblast/smooth muscle cluster (C8 and C10, Figs 5B,C and 9A). Cluster 10 is a distinct population of cells specifically appearing after injury that, in addition to the endocardial and thrombocyte populations, strongly expresses both *myh11a* as well as collagens (Fig. 9B). The high levels of expression of smooth muscle genes, as well as collagens, suggest that these cells are mainly myofibroblasts, but may also include smooth muscle cells. In the uninjured wild-type heart, only a few cells from both populations were positive for Runx1-Citrine (Figs 1A and 9A), but there was heart-wide activation of Runx1 in the epicardium and myofibroblasts after injury (Figs 1B-C, 5E and 9A). The epicardial cell cluster expressed a combination of genes known to be epicardial specific, including *tcf21* and *wt1a/b*, while *myh11a* expression was specific for the myofibroblast cluster. However, other myofibroblast genes, such as *tagln* and collagens, were present in both the epicardial and myofibroblast clusters (Fig. 9B). Staining on sections confirmed the absence of Myh11, but presence of Tagln in the epicardium (Fig. 9C). In the *runx1* mutant, similar to the reduction in other *myh11a*-expressing cells types, the number of myofibroblasts was much lower. In contrast, the epicardial cell population was slightly larger in the mutant (Fig. 9D), but had reduced levels of both collagen and smooth muscle genes (Fig. 9E). The location of the myofibroblast C10 cluster on the UMAP plot in Fig. 5B, as well as the presence of *tbx18* and *tcf21* in this cluster, suggest that these cells are more closely related to the epicardial cluster than to the *myh11a*-expressing endocardial cells. The presence of myofibroblast genes in the epicardium, and vice versa, might reflect the lineage transition from epicardial cells to myofibroblasts that has been shown before (González-Rosa et al., 2012). Both the C8 and C10 cluster express genes involved in EMT, which are reduced in the mutant, possibly suggesting reduced EMT from the epicardium. Analysis on sections confirmed that Myh11-positive cells close to the epicardium were less abundant in the mutant compared with the wild type (arrowheads, Fig. 9F,G). The strong reduction in myofibroblasts, in addition to the absence of smooth muscle gene-expressing endocardium and thrombocytes, further explains the difference in wound composition between the mutant and wild type.

### Upregulation of plasminogen receptor annexin A2 in the *runx1* mutant myocardium and endocardium

**Fig. 10.**
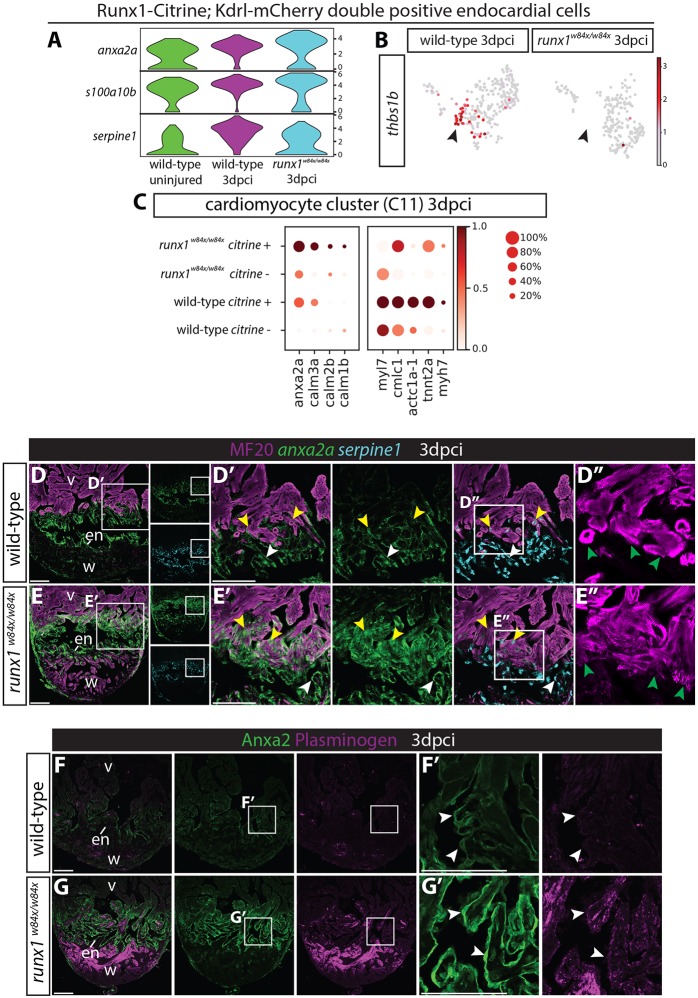
***Runx1* mutant hearts upregulate Anxa2 and plasminogen.** (A) Violin plots showing upregulation of *anx2a* and *s100a10b* in wild-type *runx1/citrine;mcherry/kdrl* double-positive cells after injury, with even higher expression in the *runx1* mutant. In contrast, *serpine1* is downregulated in the mutant cells. (B) UMAP plot of the *runx1/citrine;mcherry/kdrl* double-positive cells showing *thbs1b*-expressing cells. Arrowheads indicate *thbs1b* expression mainly in cluster 4 from Fig. 6A, which is missing in the *runx1* mutant after injury. (C) Dotplot showing that *anxa2a*, *calm1b*, *calm2b* and *calm3a* are upregulated in the mutant citrine-positive myocardium at 3 dpci, whereas sarcomere genes are upregulated in the wild-type citrine-positive myocardium. (D-E′) Section *in situ* hybridisation for *anxa2a* and *serpine1* with immunohistochemistry for MF20 shows that *anxa2a* has a similar expression pattern to Runx1-Citrine after injury in the wild type, but is expressed at much higher levels in the mutant endocardium (white arrowheads) and myocardium (yellow arrowheads). *serpine1* expression is found in both the wound border endocardium (white arrowheads) and myocardium (yellow arrowheads). (D″,E″) Sarcomere structure differs between the wild-type and the mutant in the wound border (green arrowheads). (F-G′) Immunohistochemistry for plasminogen and Anxa2 shows upregulation of plasminogen in the area where Anxa2 is upregulated (arrowheads) in the mutant. en, endocardium; v, ventricle; w, wound. Scale bars: 100 μm.

In addition to smooth muscle genes, our single cell sequencing data showed upregulation of a number of genes involved in fibrinolysis in the endocardium after injury, including *anxa2a* and *s100a10b* (Fig. 10A). Anxa2 is a calcium-dependent phospholipid-binding protein that forms the annexin A2 hetero-tetramer protein complex together with S100A10 (Madureira et al., 2011) and is an important plasminogen receptor. Plasminogen is required for dissolving fibrin blood clots and acts as an important protease in tissue remodelling and repair. The annexin A2 complex was specifically upregulated after injury in the wild-type *runx1-citrine*-positive endocardium, but much stronger in the *runx1-citrine*-positive *runx1* mutant endocardium (Fig. 10A). In contrast, *serpine1* (also called plasminogen activator inhibitor 1), an inhibitor of fibrinolysis, was much more strongly upregulated in the injured wild-type endocardium than in the *runx1* mutant (Fig. 10A), suggesting that increased fibrinolysis in the *runx1* mutant underlies the reduced amount of fibrin in the wound (Fig. 2). Interestingly, *thbs1b* (thrombospondin 1b), which is required for thrombocyte and fibrin aggregation, is specifically expressed in the smooth muscle-expressing endothelial population that is missing in the mutant, as a potential link to the absence of thrombocytes and fibrin aggregates in the mutant (arrowheads, Fig. 10B).

In addition to its expression in the endocardium, we also found calcium-dependent *anxa2a* to be the most-upregulated gene in the myocardium in the *runx1* mutant, again suggesting an increased expression of the plasminogen receptor in the mutant compared with wild types (Fig. 10C). In a previous study, cardiomyocyte specific knockout of Runx1 in the mouse showed increased sarcoplasmic reticulum calcium content and sarcoplasmic reticulum-mediated calcium release in the myocardium after injury (McCarroll et al., 2018). Correspondingly, we found increased expression of calcium-responsive genes in the Citrine-positive myocardium of our zebrafish *runx1* mutant after injury; including calmodulins *calm1b*, *calm2b* and *calm3a* (Fig. 10C, cluster C11 in Fig. 5B,C). We also observed increased expression of genes important for sarcomere formation in the Citrine-positive myocardium in the wild-type hearts (Fig. 10C). Border zone cardiomyocytes have been shown to undergo de-differentiation with loss of sarcomere structures and re-expression of embryonic myosin, followed by re-differentiation and sarcomere formation (Jopling et al., 2010; Sallin et al., 2015; Wu et al., 2016). In line with this, we also found upregulation of embryonic myosin *myh7* in the wild-type Citrine-positive cardiomyocytes (Fig. 10C). The combination of embryonic and mature myosin RNA expression points to these cells acquiring a more embryonic state, while undergoing the de- and re-differentiation process during proliferation. In the *runx1* mutant, however, this upregulation of both embryonic and mature sarcomere genes was reduced at the mRNA level (Fig. 10C). This could potentially be explained by the much higher rate of proliferation in the *runx1* mutant, resulting in a prolonged de-differentiation state before specific activation of myosin/sarcomere gene expression or alternatively, the presence of surviving cardiomyocytes in this cluster. The wound border cardiomyocytes in the mutant show a less organised structure and include surviving cells that have lost their sarcomere structure (Fig. 10D-E″).

We next confirmed the expression of both *anxa2a* and *serpine1* at 3 dpci. *anxa2a*, as well as Anxa2 protein, has a very similar expression pattern to *runx1* but with much stronger expression in the endocardium (white arrowheads) and myocardium (yellow arrowheads) in the mutant than in the wild type (Fig. 10D-G′). *serpine1* was indeed also expressed in the wound border myocardium and endocardium, further confirming the single cell data. The increase in *anxa2a* and *s100a10b*, which together convert plasminogen into plasmin to increase fibrinolysis, accompanied by the reduction in the fibrinolysis inhibitor *serpine1*, all point to substantial differences in plasmin levels and thus fibrinolysis in the *runx1* mutant. In concordance, plasminogen was far more abundant in the wound in the mutant (Fig. 10F,G). Therefore, in addition to a lack of fibrin deposition by endocardial cells, thrombocytes and myofibroblasts, *runx1* mutant hearts also show increased fibrinolysis that further explains the observed reduced fibrin presence in the wound and the faster repair in the *runx1* mutant.

## DISCUSSION

By interrogating Runx1 function at the single cell level in a global *runx1* null mutant, we have exposed Runx1 as an inhibitor of heart repair on many levels. The overarching change we observed in different injury-responsive cell types was a strong reduction of smooth muscle and collagen gene expression in the mutant after injury. The expression of high levels of smooth muscle and collagen genes is a hallmark of myofibroblasts, but we identified both endocardial cells and thrombocytes as expressing myofibroblast-like genes after injury. The endocardium proximal to the wound has been described previously to upregulate collagens (Münch et al., 2017; Sánchez-Iranzo et al., 2018) as well as smooth muscle genes such as *tagln* and *myl6* (Wu et al., 2016). However, the thrombocytes that make up a large proportion of the wound have not yet been described to express Myh11. Surprisingly, we observed the most collagen deposition in the wound localised adjacent to the Myh11-expressing endocardial cells and thrombocytes, and to a much lesser extent near the epicardium, despite the fact that the epicardium is considered the main source of myofibroblasts and collagen deposition in the heart (González-Rosa et al., 2012; Sánchez-Iranzo et al., 2018). This suggests that, although these cells retain the characteristics of endocardial cells and thrombocytes, they can function analogously to myofibroblasts. The double identity of these cells may mean that the cells responsible for the injury-induced fibrosis in fish are more transient and less differentiated compared with fully mature myofibroblasts. This in turn may reflect deposition of less stable, degradable fibrotic tissue compared with the mammalian situation, in which myofibroblasts predominate and a scar can persist for many years after myocardial infarction (Turner and Porter, 2013). *runx1* mutants do not have a Myh11-expressing endocardial and thrombocyte population, and have fewer myofibroblasts. This alone could explain the significant reduction in collagen and fibrin in the wound; however, the *runx1* mutant also shows increased expression of the plasminogen receptor annexin A2 as well as plasminogen itself. Annexin A2 converts plasminogen into plasmin, the major fibrinolytic agent that breaks down fibrin in blood clots (Madureira et al., 2011; Christensen et al., 2018). In addition to a reduction in collagen- and fibrin-depositing cells, the increased levels of plasminogen point to faster degradation of deposited fibrotic tissue, allowing improved migration of myocardial cells into the wound to regenerate the heart.

The *Tg(BAC-runx1P2:Citrine)* reporter line we used was made using bacterial artificial chromosome technology (BAC) to create a transgene that has both *runx1* promoters, including a large upstream region. The BAC contains the *runx1* P1 promoter region with ∼25,000 bp of upstream sequence and the P2 promoter region (with a ∼90,000 bp large intron between P1 and P2), and contains only exon 2b and 3, but none of the other exons further downstream (Bonkhofer et al., 2019). The inclusion of the very large upstream regulatory region of *runx1* likely explains the observed fluorescent transgene expression differences when compared with any other previously published *runx1* transgenic fish lines (Jin et al., 2009; Goldman et al., 2017). Goldman et al. have identified a 103 kb upstream region of *runx1* that specifically drives expression in the zebrafish wound border myocardium after injury, which they called a ‘cardiomyocyte regeneration enhancer’ (CREE) (Goldman et al., 2017). The expectation, based on the results with this enhancer, was that Runx1 expression in the myocardium near the wound is beneficial for heart regeneration. A positive role for Runx1 was also suggested by its upregulation in hearts treated with oncostatin M, which has been shown to protect the heart after acute myocardial infarction (Kubin et al., 2011), as well as in hearts that overexpress Erbb2 and show enhanced myocardial proliferation and regeneration (D'Uva et al., 2015). High levels of cardiomyocyte Runx1 expression were linked to the reduced differentiation state of cardiomyocytes in these models, which facilitates the dedifferentiation and increased proliferation that lead to improved levels of heart repair. However, this hypothesis was solely based on expression data and not functionally tested. In contrast, our findings demonstrate the opposite: loss of *runx1* results in enhanced heart regeneration, by increasing myocardial proliferation, increasing myocardial survival and altering wound tissue composition, as discussed above. This poses the question of why Runx1 is specifically upregulated during both zebrafish heart regeneration and mammalian heart repair (Kubin et al., 2011; D'Uva et al., 2015; Goldman et al., 2017; McCarroll et al., 2018) when it seems to function to inhibit key regenerative processes? The answer might lie in the fact that absence of *runx1* causes increased proliferation and upregulation of the annexin 2a receptor, which is strongly linked to proliferation in cancer (Christensen et al., 2018). Runx1 might function to keep proliferation of cardiomyocytes, as well as other cell types, in check and to prevent them from growing out of control during myocardial regeneration. This fits well with the observations that Runx1 acts as a key factor in determining the proliferative and differential states of multiple cell types (Murthy et al., 2014; Umansky et al., 2015; Bao et al., 2018; Sarper et al., 2018; Zhou et al., 2018) alongside different functions that correlate with levels of Runx1 expression (Lie-a-ling et al., 2018; Antony-Debré et al., 2019), with higher levels shown to result in cell fate transition and differentiation (Lee et al., 2014).

Taken together, our data suggest that Runx1 functions to regulate scar deposition and degradation, and to repress myocardial proliferation and differentiation, as well as myocardial survival in the zebrafish heart. The fact that one gene can inhibit multiple aspects of heart regeneration offers the exciting prospect that all these processes can be targeted simultaneously in efforts to achieve human heart repair. Of note, small molecule drugs inhibiting Runx1 have already passed pre-clinical testing in the context of leukaemia treatment (Illendula et al., 2016). Even though the zebrafish is capable of regeneration, the *runx1* mutant shows that this process may not be optimal, arising from the need to initiate a fibrotic response for immediate repair and to potentially keep cardiomyocyte proliferation under control during myocardial regeneration. During evolution, adult zebrafish seem to have established a fine balance between regulating fibrosis and myocardial proliferation without losing control of cell division; understanding how this balance is maintained may open up novel targets for future therapeutic interventions.

## MATERIALS AND METHODS

### Zebrafish strains and husbandry

All experiments were carried out under appropriate Home Office licences and in compliance with the revised Animals (Scientific Procedures) Act 1986 in the UK and Directive 2010/63/EU in Europe, and all have been approved by Oxford University's central Committee on Animal Care and Ethical Review (ACER). Adult wild-type (wt) (KCL strain), *Tg(kdrl:Hsa.HRAS-mCherry)* (Illendula et al., 2016; Chi et al., 2008), *runx1^W84X^* mutants (Jin et al., 2012) and *TgBAC(runx1P2:Citrine)* (Bonkhofer et al., 2019) were housed in a Techniplast aquarium system (28°C, 14/10 h light/dark cycle, fed three times daily with dry food and brine shrimp). All double transgenic lines on a wild-type or mutant background were generated by natural mating.

### Cardiac surgery

All procedures/protocols were carried out in accordance with UK Home Office regulations, with respective project licences held in all contributing labs, approved by Home Office inspectors and local representatives. Zebrafish cryo-injury and resection injury of the ventricle were performed as previously reported (González-Rosa and Mercader, 2012; Koth et al., 2017). Briefly, prior to all surgical operations, fish were anaesthetised in MS222 (Sigma). A small incision was made through the thorax and the pericardium using forceps and spring scissors. The abdomen was gently squeezed to expose the ventricle and tissue paper was used to dry the heart. A cryo-probe with a copper filament was cooled in liquid nitrogen and placed on the ventricle surface until thawing was observed. Body wall incisions were not sutured, and after surgery, fish were returned to water and stimulated to breathe by pipetting water over the gills until they started swimming again. For sham surgery, the thorax and pericardial sac were opened, and the heart was touched, but not injured. All operated fish were kept in individual tanks for the first week after surgery, then fish were combined in larger tanks. The surgeries were carried out at the same time during the day for all groups, and by the same person to keep variation to a minimum.

### Tissue processing

Hearts were extracted and transferred to Ringer solution with heparin sodium salt (50 U/ml) (Ringer composition: 7.2 g NaCl, 0.225 g CaCl_2_.H_2_O, 0.37 g KCl, 0.2175 g Na_2_HPO_4_.7H_2_O, 0.02 g KH_2_PO_4_ at pH 7.4 sterilised using a 0.22 μm bottle top filter unit) and rinsed once with phosphate-buffered saline (PBS) or directly isolated in PBS. Hearts were inspected, cleaned and then fixed with 4% paraformaldehyde (PFA) overnight at room temperature. Samples were rinsed once in PBS, dehydrated in ethanol at 70%, 80%, 90% and 96% for 2 h per step and twice in 100% ethanol for 1 h each step, followed by a 100% 1-butanol step overnight. The samples were then transferred to paraffin (Paraplast Plus, Sigma-Aldrich, P3683) wax at 65°C. Paraffin was refreshed twice with each step taking at least 2 h, prior to mounting in a sectioning mould. Sections (10 µm) were cut using a Leica microtome and section ribbons were stored on black cardboard in shallow stackable plastic trays. Individual sections, evenly distributed throughout the heart, e.g. 1 in 6 sections, were selected and mounted on superfrost plus glass slides for histology, histochemistry and RNA labelling (RNAscope).

### Histology

For Acid Fuchsin Orange G-staining (AFOG), dewaxed and water-rinsed sections were refixed in Bouin's solution for 3 h at 60°C and then washed in double distilled H_2_O until sections were white/clear, incubated in aqueous 1% phosphomolybdic acid for 5 min, rinsed with double distilled  H_2_O, stained with AFOG solution for 5 min, rinsed briefly in double distilled H_2_O, dehydrated quickly through 70%, 80%, 90%, 96% and 100% ethanol, cleared in Xylene and mounted in DPX mounting medium. To make AFOG solution, boil 1 l double distilled H_2_O with 5 g of Methyl Blue (Sigma, 95290). Once cooled add 10 g Orange G (Sigma, O7252) and 15 g Acid Fuchsin (Sigma, F8129), and adjust to pH 1.09 by adding HCl (at 25% concentration).

### Immunohistochemistry

Fluorescent immunohistochemistry was performed as previously described (Mommersteeg et al., 2010). For de-waxing, slides were taken through Xylene twice for 5 min, 100% ethanol twice for 1 min, then once for 1 min in each of 96%, 90%, 80% and 70% ethanol, with a final rinse in PBS-T prior to subsequent staining. De-waxed and rehydrated sections were heated up and then pressure cooked for 4 min in antigen unmasking solution (H-3300, Vector Laboratories). Once cooled, sections were placed in PBS before drawing a ring (ImmEdge pen, Vector Laboratories) around the sections. Slides were placed into staining trays providing humidity and blocked using TNB [0.5% TSA blocking reagent, 0.15 M NaCl, 0.1 M Tris-HCl (pH 7.5), NEL702001KT, Perkin Elmer] for 30 min at room temperature. Blocking agent was removed and primary antibody in TNB was added and incubated overnight at room temperature. Slides were then washed three times for 5 min in PBS before the secondary antibody (Alexa range, Invitrogen) at 1:200 dilution in TNB was added for 2 h at room temperature. For some primary antibodies, an additional amplification step was added to enhance the signal using the TSA kit (NEL756001KT, Perkin and Elmer). Instead of an Alexa secondary antibody, a biotinylated secondary antibody was used at a 1:200 dilution in TNB for 45 min at room temperature, followed by three 5 min washes in PBS-T prior to a 30 min incubation with conjugated Streptavidin-Horse Radish Peroxidase (Vector Laboratories, SA-5004) and then three 5 min washes in PBS-T. Either fluorescein or tetramethylrhodamine (in DMSO) diluted at 1:100 in amplification buffer was then added to the sections for 3 min, followed by three 5 min washes with PBS and staining with DAPI (2.5 µg/ml, Sigma). Slides were mounted in Mowiol 4-88 (Applichem) and incubated at 37°C overnight in the dark. Primary antibodies against the following proteins were used: chicken polyclonal against green fluorescent protein (GFP, 1:200, Aves Lab, GFP-1020), mouse monoclonal against mCherry (clone 1C51, 1:200, Abcam, ab125096), proliferating cell nuclear antigen (PCNA, clone PC10, 1:200, Dako Cytomation, M0879), myosin heavy chain (MF20, 1:50, HSHB AB-2147781), plasminogen (Plg, 1:200, R&D systems, MAB1939), rabbit polyclonal against lysozyme (LyC, 1:200, Anaspec, AS-55633), ETS transcription factor ERG (ERG, 1:200, Abcam, ab110639), myocyte enhancer factor 2 (Mef2 C-21, 1:200, Santa Cruz, sc-313), smooth muscle myosin heavy chain 11 (Myh11, 1:200, Abcam, ab125884), transgelin (SM22a, 1:200, Abcam, ab14106) and annexin A2 (Anxa2, 1:200, Invitrogen, PA5-14317). Most antibodies used were those commonly used in zebrafish research. Myh11 and annexin A2 were validated by overlapping expression with the respective RNAscope probes. For double labelling with RNAscope probes, RNAscope was performed first and then samples were processed for immunohistochemistry as described above, starting from the blocking step. Images were processed using ImageJ to generate magenta and green colour combinations.

### RNAscope *in situ* hybridisation

RNAscope (Advanced Cell Diagnostics, Hayward, CA) (Wang et al., 2012) was performed on 10 µm paraffin sections, processed as described above. Sections were baked at 60°C for 1 h before deparaffinisation using two 5 min Xylene washes followed by two 2 min washes in 10% ethanol. The slides were air-dried followed by incubation in RNAscope Hydrogen Peroxide (H_2_O_2_) for 15 min before washing in MilliQ water. The slides were then boiled at 98-102°C for 15 min in 1× RNAscope Target Retrieval solution, placed in 100% ethanol for 3 min and air dried. The sections were then incubated with RNAScope Protease III in a Hybez oven at 40°C for 12 min, washed in MilliQ twice for 2 min, followed by incubation with the different RNAScope probes for 2 h at 40°C. The RNAscope Multiplex Fluorescent Detection Reagents v2 and the TSA Plus Cyanine 3 and 5 fluorophore (Perkin Elmer, NEL744001KT) were applied according to the manufacturer's instructions. The slides were further processed for immunohistochemistry or mounted in Mowiol 4-88. Advanced Cell Diagnostics designed the probes. Probes used were Dr-tcf21-C2 (485341-C2), Dr-itga2b-C2 (555601-C2), Dr-runx1 (433351), Dr-myb-C3 (558291-C3), Dr-gata2b-C2 (551191-C2), Dr-anxa2a (587021) and Dr-serpine1 (551171-C3).

### Image acquisition and data analysis

Images were acquired using either a Zeiss LSM880 or Olympus FV3000 confocal microscope. Images were processed in FIJI/ ImageJ to generate a magenta/green/cyan/grey colour scheme. For all quantifications on sections, individual sections were mounted, evenly distributed throughout the heart, e.g. 1 in 6 sections, to reduce the number of hearts needed and guarantee even coverage of the entire heart. Using Fiji/image J, myocardial regeneration was then quantified by measuring the perimeter of the ventricle of each heart section of AFOG-stained hearts and the length of open compact myocardium. The biggest open myocardium length and ventricle perimeter measurement from each fish were then taken and the open myocardium length was divided by the ventricle perimeter and multiplied by 100 to calculate the percentage of the myocardium that was still open. Wound area and ventricle area were also measured for each section (using Fiji/image J) and again the biggest measurement of each for each fish was used to calculate the size of the wound region. This was achieved by dividing the wound area by the ventricle size, then multiplying by 100 to give a percentage. For analysis of the colour of the wound area on the sections stained with AFOG, we split the colour photo of the wound area up into a red, green and blue channel. The images were then thresholded using the same settings for all hearts for the red and blue channel. The orange area was determined by subtracting the red and blue areas from the total area.

Myocardial proliferation was assessed by using Mef2, a nuclear myocardial marker, and proliferating cell nuclear antigen (PCNA), a nuclear marker of proliferation, on antibody stained sections. The border zone region was established as the cardiomyocytes closest to the wound in the healthy myocardial tissue. The number of Mef2^+^ nuclei was counted and the number of PCNA/Mef2 double^+^ nuclei counted and their percentage calculated for at least three sections per fish.

### Heart processing for FACS

Freshly isolated hearts were placed in chilled Hanks balanced buffered saline (HBBS). The atria and bulbus arteriosus were removed, and the ventricle was cut into several pieces using fine forceps and ophthalmic scissors (FST, 15009-08). The following digestion procedure was adapted from Cao et al. (2015). Pieces of tissue were transferred to a 2 ml tube, rinsed with HBBS and 1 ml (up to 10 hearts) of digestion mix (0.13 U/ml Liberase DH (Roche). 1% sheep serum in HBBS was added and the sample incubated at 32°C under 80-100 rpm rotation/agitation. Every 10-15 min, supernatant was collected and placed on ice, a new digestion mix was added, and tissue and solution were gently pipetted 5-10 times to aid break up. Once all the tissue was resolved (∼1 h), all collected suspensions were spun at 300 ***g*** for 10 min. Supernatant was removed and replaced with 1% fetal bovine serum in HBBS, and suspensions were then combined and placed on ice. Prior to FACS (MoFlo Asterios, Beckman and Coulter), cells were stained with DAPI to discard dead cells during the sort.

### Single cell sequencing

Cells from 20 hearts per sample from isolated uninjured wild-type and 3 dpi injured wild-type and *Runx1^W84X/W84X^* ventricles were FACS sorted, and populations of single- and double-positive cells were isolated separately. The single- and double-positive cells were then mixed, so that the samples were one-third Citrine positive, one-third double positive and one-third mCherry positive. Cells were washed in PBS with 0.04% BSA and re-suspended before loading 12,000-12,500 cells onto each channel of the Chromium 10x Genomics platform to capture single cells in droplets. Library generation for 10x Genomics v2 chemistry was performed following the Chromium Single Cell 3′ Reagents Kits User Guide CG00052. Quantification of cDNA was performed using a Qubit dsDNA HS Assay Kit (Life Technologies, Q32851) and a high-sensitivity DNA tape-station (Agilent, 5067-5584). Quantification of library construction was performed using a Qubit dsDNA HS Assay Kit (Life Technologies, Q32851) and a high-sensitivity DNA tape-station (Agilent, 5067-5584). Libraries were sequenced on an Illumina HiSeq4000 platform to achieve a minimum of 20,000 reads per cell.

### Data analysis

#### Alignment

The count function in 10x Genomics Cellranger software (v2.1.1) was used for sample demultiplexing, barcode processing and gene counting with --chemistry=threeprime (https://support.10xgenomics.com/single-cell-gene-expression/software/pipelines/latest/installation). The Danio_rerio.GRCz11 (release 94) version of the zebrafish genome and gene annotation files used for alignment were downloaded from the Ensembl database (ensembl.org). We added the sequences for the mCherry-plasmid and Citrine-plasmid. The mCherry plasmid had three sequence contents: mCherry, the mCherry-plasmid-backbone and mCherry-polyA. The Citrine-plasmid had seven sequence contents: Citrine-3x-HA-tag, Citrine-BirA, Citrine-Tav-2a, Citrine, Citrine-polyA, Citrine-Frt1 and the remaining plasmid backbone sequences (Citrine-Remaining). The new reference with the additional sequences was built using the mkgtf function in Cellranger. In order to eliminate potential reads that were aligned to both plasmids, only uniquely mappable reads were considered for STAR alignments with an additional parameter –‘outFilterMultimapNmax’, ‘1’, added in the reference.py file.

#### Quality control

Quality control was performed from the raw counts with all barcodes. First, cells with fewer than 100 genes expressed were removed. 4720, 4754 and 6268 cells passed for the wild-type uninjured, wild-type 3 dpci and *runx1^W84X/W84X^* 3 dpci samples, respectively. Second, doublets were filtered using the scrublet (Wolock et al., 2019) package in Python. Cells with doublet score larger than 0.3, 0.27 and 0.38 for the wild-type uninjured, wild-type 3 dpci and *runx1^W84X/W84X^* 3 dpci samples, respectively were removed. This excluded 55 wild-type uninjured cells, 58 wild-type 3 dpci cells and 214 *runx1^W84X/W84X^* 3 dpci cells. After quality control, 4665 wild-type uninjured cells, 4696 wild-type 3 dpci cells and 6054 *runx1^W84X/W84X^* 3 dpci cells were retained. Mean reads per cell: 18,082 for wild-type uninjured, 18,652 for wild-type 3 dpci and 13,099 for *runx1^W84X/W84X^* 3 dpci cells, with sequencing saturation ≥50%. Non-expressed genes were removed and cells were normalized to 10,000 for each cell and log-transformed.

#### Defining cell-types based on marker genes

*mCherry*-positive (mChr^+^) cells were defined as a union of cells that have at least one unique molecular identifier (UMI) count in either *mCherry*, *mCherry-plasmid-backbone* or *mCherry-polyA*. Citrine positive (Cit^+^) cells were defined as a union of cells that have at least 1 UMI in either *citrine*, *citrine-polyA* or *citrine-remaining*. If the cell had at least 1 UMI for *kdrl/runx1*, then the cell was labelled as *kdrl* positive (*kdrl*^+^) or *runx1* positive (*runx1*^+^). *kdrl*^+^*mChr*^+^ cells were defined as the cells that were either *kdrl^+^* or *mChr^+^*. Runx1^+^Cit^+^ cells were defined as the cells that are either *runx1^+^* or *cit^+^*. Finally, the double-positive (double^+^) cells were defined as cells that are both *kdrl^+^mChr^+^* and *runx1^+^cit^+^*. The number of cells for each cell type is summarised in Table 1. For extracting subsets of cells, including cardiomyocytes (255 cells), double^+^ cells (561 cells) and double^+^ without *runx1^W84X/W84X^* cells (361 cells), the same quality control pipeline was applied.

**Table 1.**
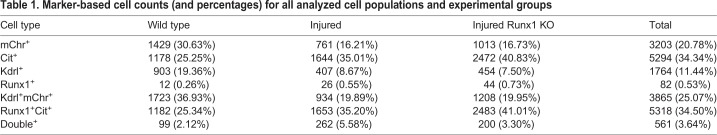
**Marker-based cell counts (and percentages) for all analyzed cell populations and experimental groups**

#### Selection of highly variable genes

Highly variable genes (HVGs) were selected following the Seurat method (Butler et al., 2018) (a method with parameters min_mean=0.0125, max_mean=4 and min_disp=0.5). HVGs (4663, 2341, 3484 and 3377) were selected for all cells, for CMs, for double^+^ cells and for double^+^ without injured *runx1^W84X/W84X^* cells. Cells were then log-transformed. The effects of total number of counts and the percentage of mitochondrial genes were regressed out and each gene was scaled so that it was zero-centred.

#### Visualization and clustering

For UMAP visualisation (Uniform Manifold Approximation and Projection), first, a k=10 nearest neighbour graph was calculated on the first 50 principal components of the PCA, based on HVGs using the neighbours function in Scanpy (Wolf et al., 2018). The UMAP was then calculated based on this k-nearest-neighbour graph using the UMAP function in Scanpy. The sub cell populations were determined by Louvain clustering with resolution 1, 0.5, 0.6, 0.7 for all cells, cardiomyocytes, double^+^ cells and double^+^ without *runx1^W84X/W84X^* cells, respectively. In total, 26 clusters were defined in all cells, three clusters for CMs and five clusters for both double^+^ cells and double^+^ without *runx1^W84X/W84X^* cells.

#### Differential expression and gene ontology annotation

Differential expression (DE) analysis was performed using rank_genes_groups in Scanpy with the ‘*t*-test_overestim_var’ method that overestimates the variance of each group. The *P* values were corrected by the ‘benjamini-hochberg’ (BH) method to account for the multiple comparison problem. Gene ontology (GO) information was downloaded from the ZFIN database (https://zfin.org/downloads). Only GO terms with more than five genes and fewer than 500 genes were considered. The enriched GO terms were calculated using a hypergeometric test on the top 50 genes using phyper function in R. The *P* values were then corrected by the ‘benjamini-hochberg’ (BH) method using the p.adjust function in R.

#### Data plotting

The violin and heatmap plots were made using seaborn and matplotlib modules in Python, and the dotplots using the dotplot function in Scanpy. The dotplots of the epicardial and the myofibroblast clusters (Fig. 8E) were coloured by the scaled mean expression value by dividing its maximum. The size of dots indicates the number of cells expressing the selected genes for each group. This number was scaled by dividing its maximum. The dotplot of the CM cluster (Fig. 9C) was coloured by the scaled mean expression value for each group, calculated by subtracting the minimum and dividing each by its maximum. The dot size was represented as the fraction of cells expressing the selected genes.

### Statistical analysis

The number of samples (*n*) used in each experiment is shown in the legends and recorded in detail below. Appropriate sample sizes were computed when the study was being designed and no data was excluded. ANOVA tests were applied when normality and equal variance tests were passed. Surgeries were not randomised, but during analysis, measurements and counts were performed blinded. Animals of the same age were used within experiments and as controls. Animals were not selected for sex. Results are expressed as mean±s.e.m. (*P<0.05, **P<0.01, ***P<0.001 and ****P<0.0001). Statistical analysis was performed in GraphPad Prism 6 for Windows (www.graphpad.com).

Fig. 2F uses two-way ANOVA with a Sidak test. For all time points, *n*=5 wild type, *n*=5 *runx1* mutants. Comparing wild type versus mutant per time point: 3 dpci, *P*=0.1951; 7 dpci, *P*=0.9486; 14 dpci, *P*=0.0034; 30 dpci, *P*>0.9999; 70 dpci, *P*=0.9973.

Fig. 2G uses two-way ANOVA with a Sidak test. For all time points, *n*=5 wild type, *n*=5 *runx1* mutants. Comparing wild type versus mutant per time point: 3 dpci, *P*=0.0222; 7 dpci, *P*=0.7108; 14 dpci, *P*=0.1220; 30 dpci, *P*>0.3548; 70 dpci, *P*=0.7223.

Fig. 2I-K uses two-way ANOVA with a Sidak test. *n*=5 wild type and *n*=5 *runx1* mutants. Orange, *P*=0.0001; red, *P*=0.0220; blue, *P*=0.1312.

Fig. 3E uses two-way ANOVA with a Sidak test. For uninjured, sham, 1 dpci and 14 dpi, *n*=4. For 3 and 7 dpci, *n*=5. Time points were compared within a wound or ventricle. For all ventricle comparisons, *P*>0.9999. For wound comparisons: uninjured versus sham, *P*=0.9954; uninjured versus 1 dpci, *P*=0.0025; uninjured versus 3 dpci, *P*=0.0005; uninjured versus 7 dpci, *P*=0.0970; uninjured versus 14 dpci, *P*>0.9999; sham versus 1 dpci, *P*=0.0501; sham versus 3 dpci, *P*=0.0144; sham versus 7 dpci, *P*=0.7469; sham versus 14 dpci, *P*>0.9999; 1 dpci versus 3 dpci, *P*>0.9999; 1 dpci versus 7 dpci, *P*=0.8847; 1 dpci versus 14 dpci, *P*=0.0118; 3 dpci versus 7 dpci, *P*=0.6164; 3 dpci versus 14 dpci, *P*=0.0029; 7 dpci versus 14 dpci, *P*=0.3351.

In Fig. 6D, data are analysed using an unpaired, two-tailed, equal variance *t*-test (*n*=5 wild type, *n*=5 *runx1* mutants; *P*=0.0239).

Fig. 10C uses two-way ANOVA with a Sidak test. For all time points, *n*=5 wild type, *n*=5 *runx1* mutants. For wild type versus mutant: 3 dpci, *P*=0.0002; 7 dpci, *P*=0.0095; 14 dpci, *P*=0.0452. Fig. 10E uses two-way ANOVA with a Sidak test. For all time points, *n*=5 wild type, *n*=5 *runx1* mutants. For wild type versus mutant: 3 dpci, *P*<0.0001; 7 dpci, *P*=0.0775.

## Supplementary Material

Supplementary information
