# Supplementary material for: Runx1 promotes scar deposition and inhibits myocardial proliferation and survival during zebrafish heart regeneration
Source: Development. 2020 Apr 27;147(8):dev186569. doi: 10.1242/dev.186569 (PMC7197712; doi:10.1242/dev.186569)
Supplement: Supplementary information [file develop-147-186569-s1.pdf]

Supplementary Figures

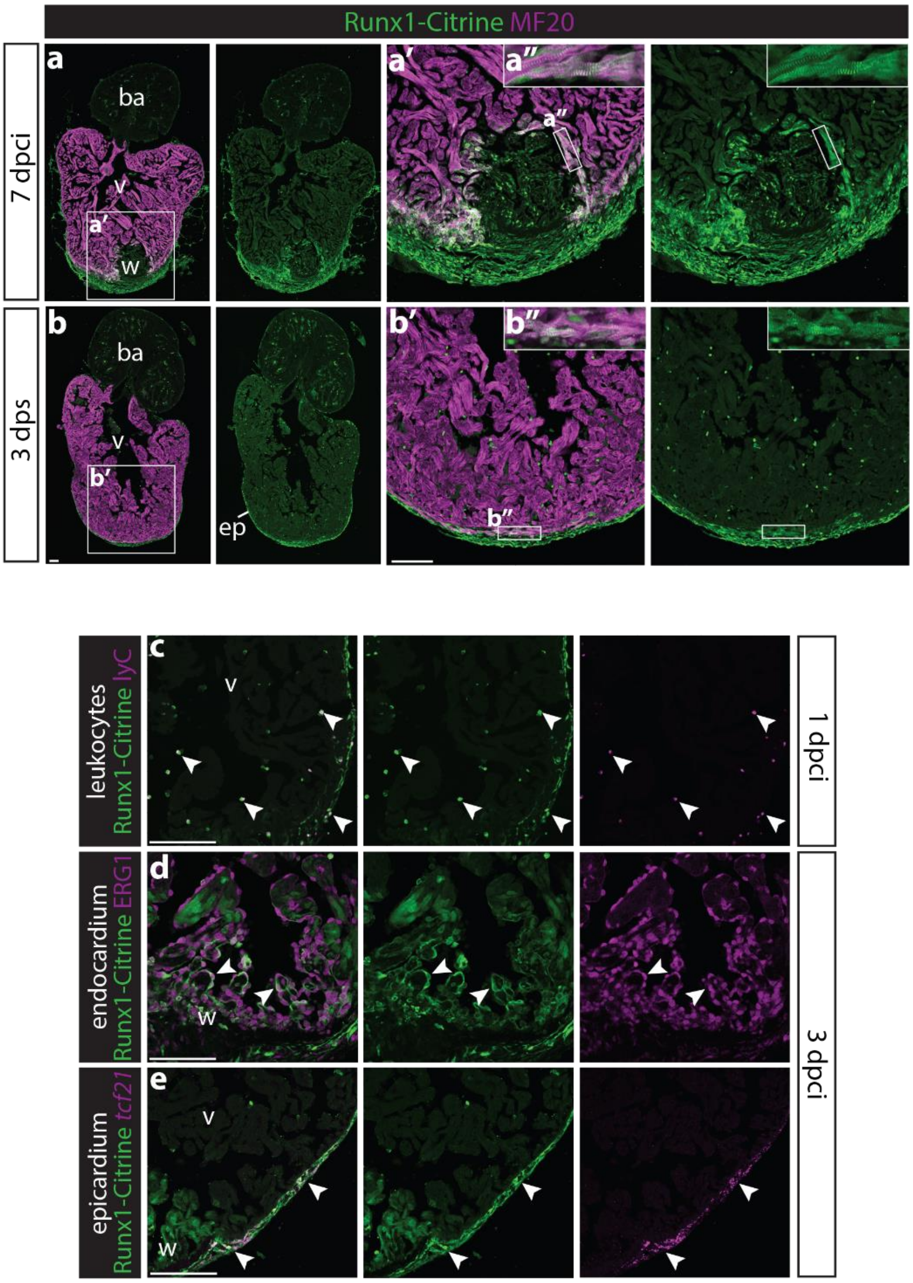

Figure S1. Runx1-Citrine becomes strongly expressed in the heart after cryo-injury.

a-b'', immunohistochemistry for Runx1-Citrine (GFP antibody) and myocardial marker MF20 at 7dpci as well as in the sham heart. a-a', at 7 dpci, the epicardium, endocardium and other wound cells were positive for Citrine. Also the myocardium in the border zone next to the wound was highly Citrine-positive (a''). b-b'', touching the heart with the probe without freezing cells and isolating the heart 3 days later (days post sham, dps) also initiates a response, with Citrine expression in the epicardium and myocardium. c-e, immunohistochemistry for Citrine, LyC, ERG1 and *in situ* hybridisation for *tcf21*. Arrowheads point to overlap of Runx1-Citrine with leukocyte marker lyC at 1dpci (c), and with endocardial marker ERG1 (d) and epicardial marker *tcf21* at 3dpci (e). a, atrium; ba, bulbus arteriosus; ep, epicardium; v, ventricle; w, wound. Scale bars depict 100 µm.

Development • Supplementary information

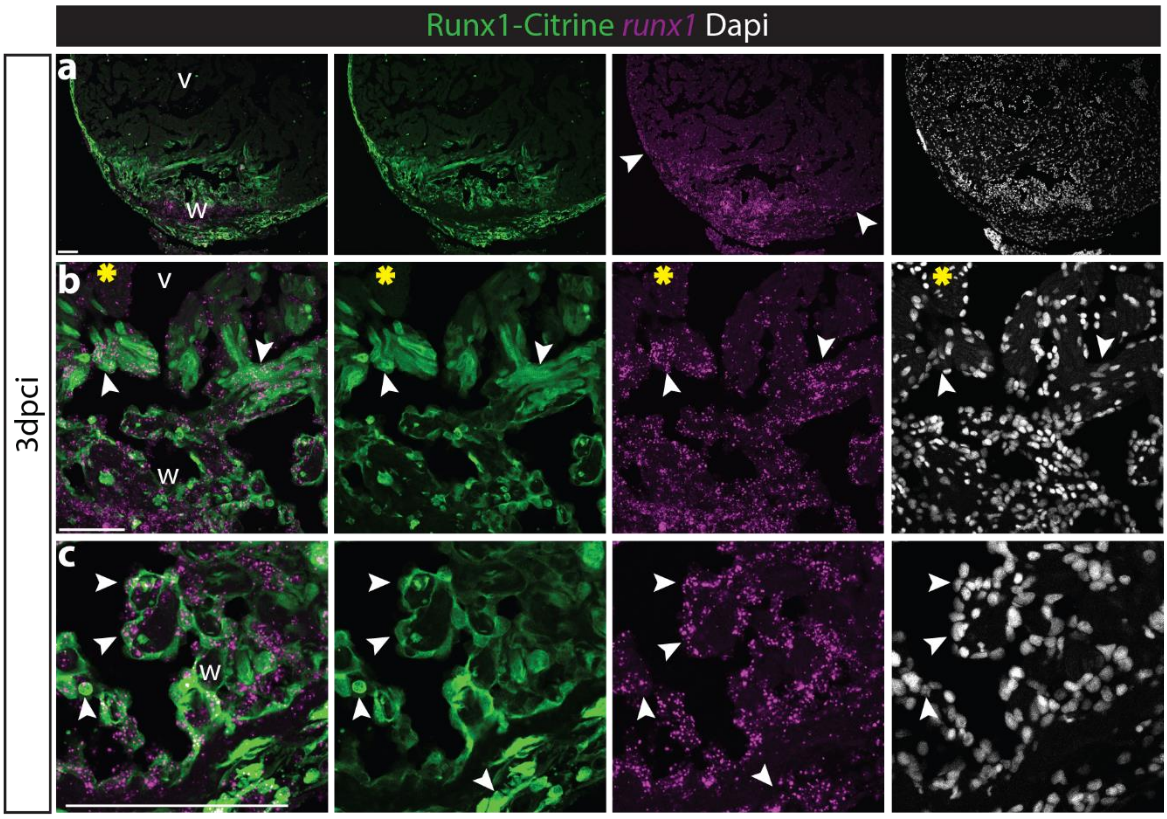

Figure S2. Runx1-Citrine expression recapitulates endogenous *runx1* expression.

a-c, immunohistochemistry for Citrine combined with *in situ* hybridisation for *runx1* showing overlap of Runx1-Citrine with runx1 mRNA in wild-type hearts. a, arrowheads point to double positive cells in the epicardium. b, arrowheads point to double positive cells in the myocardium, where the yellow asterisks point to double negative myocardium. c, arrowheads point to double positive cells in the endocardium. v, ventricle; w, wound. Scale bars depict 100 µm.

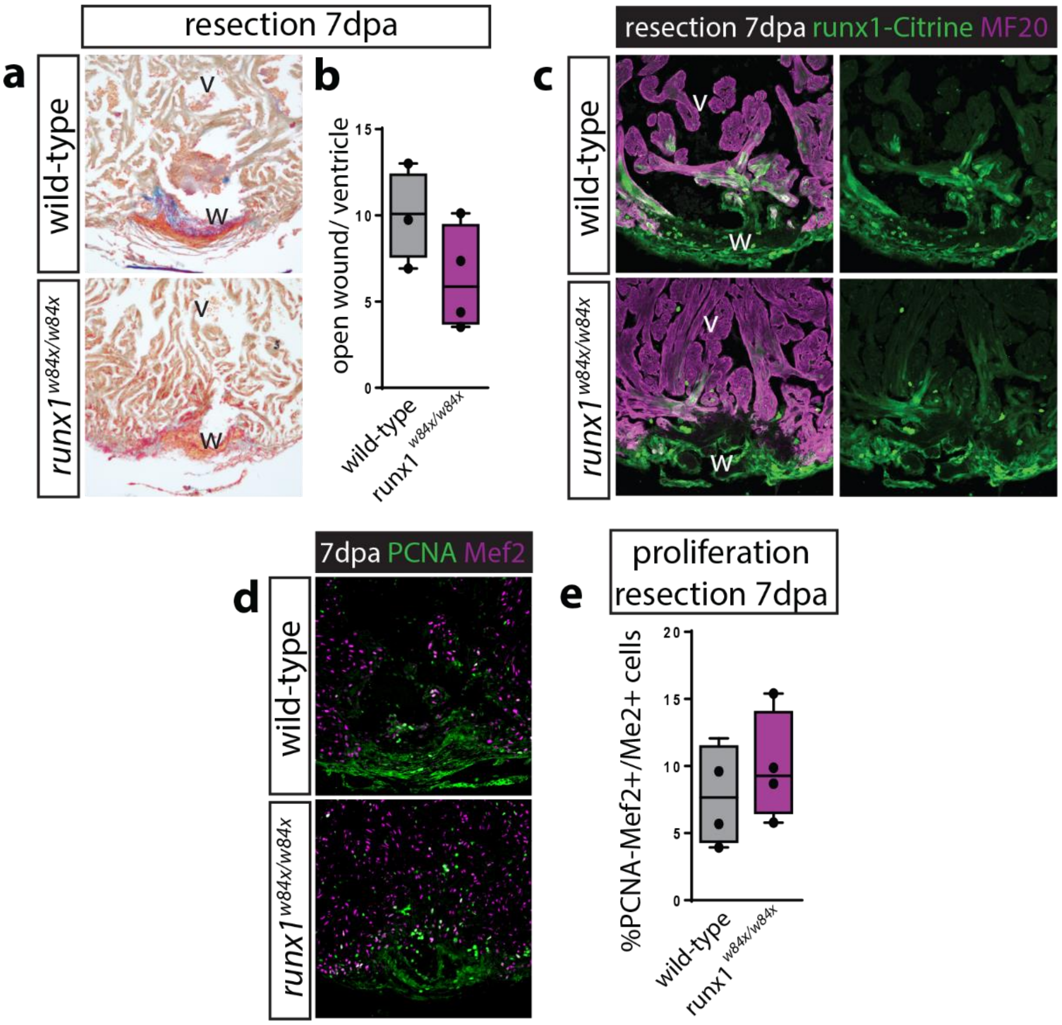

Figure S3. No significant differences in myocardial proliferation and regeneration after resection injury.

a, AFOG staining of wild-type and *runx1* mutant ventricles at 7dpa. b, immunohistochemistry for Runx1-Citrine and MF20 at different time points after resection-injury shows a similar pattern as seen after cryo-injury. d, Immunohistochemistry for PCNA and Mef2 on 7dpa (days post amputation) sections. e, quantification of PCNA-positive proliferating Mef2-positive myocardial cells after injury points to no significant differences between the *runx1* mutants and wild-types at 7dpa. n=4, two-way ANOVA with Sidak test. v, ventricle; w, wound.

Development • Supplementary information

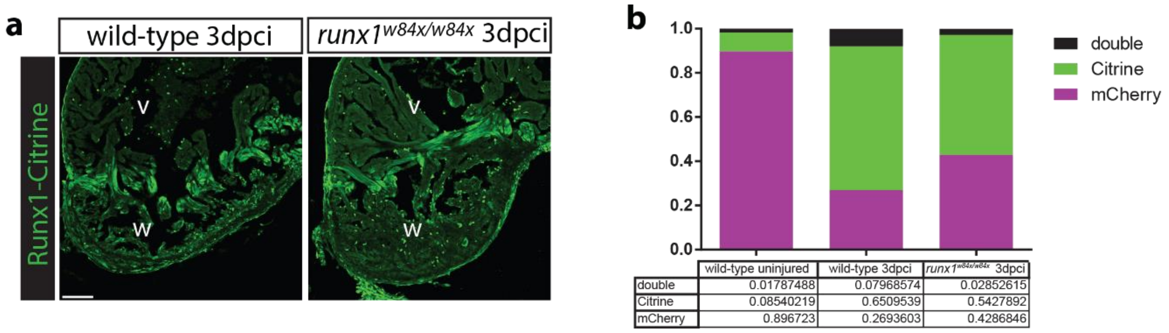

	wild-type uninjured	wild-type 3dpci	*runx1*$^{w84x/w84x}$ 3dpci
double	0.01787488	0.07968574	0.02852615
Citrine	0.08540219	0.6509539	0.5427892
mCherry	0.896723	0.2693603	0.4286846

Figure S4. Reduced number of Citrine-positive endocardial cells in the *runx1* mutant.

a, immunohistochemistry for Citrine at 3dpci. Similar expression of the Runx1-Citrine protein between wild-type and *runx1* mutant hearts after injury. b, FACS sorting for Runx1-Citrine and kdrl-mCherry shows an increase in double positive cells after injury in the wild-type compared to the uninjured wild-type hearts, whereas there is a reduction in the number of double positive cells in the injured *runx1* mutant compared to the injured wild-types. v, ventricle; w, wound. Scale bars depict 100 µm.

Development • Supplementary information

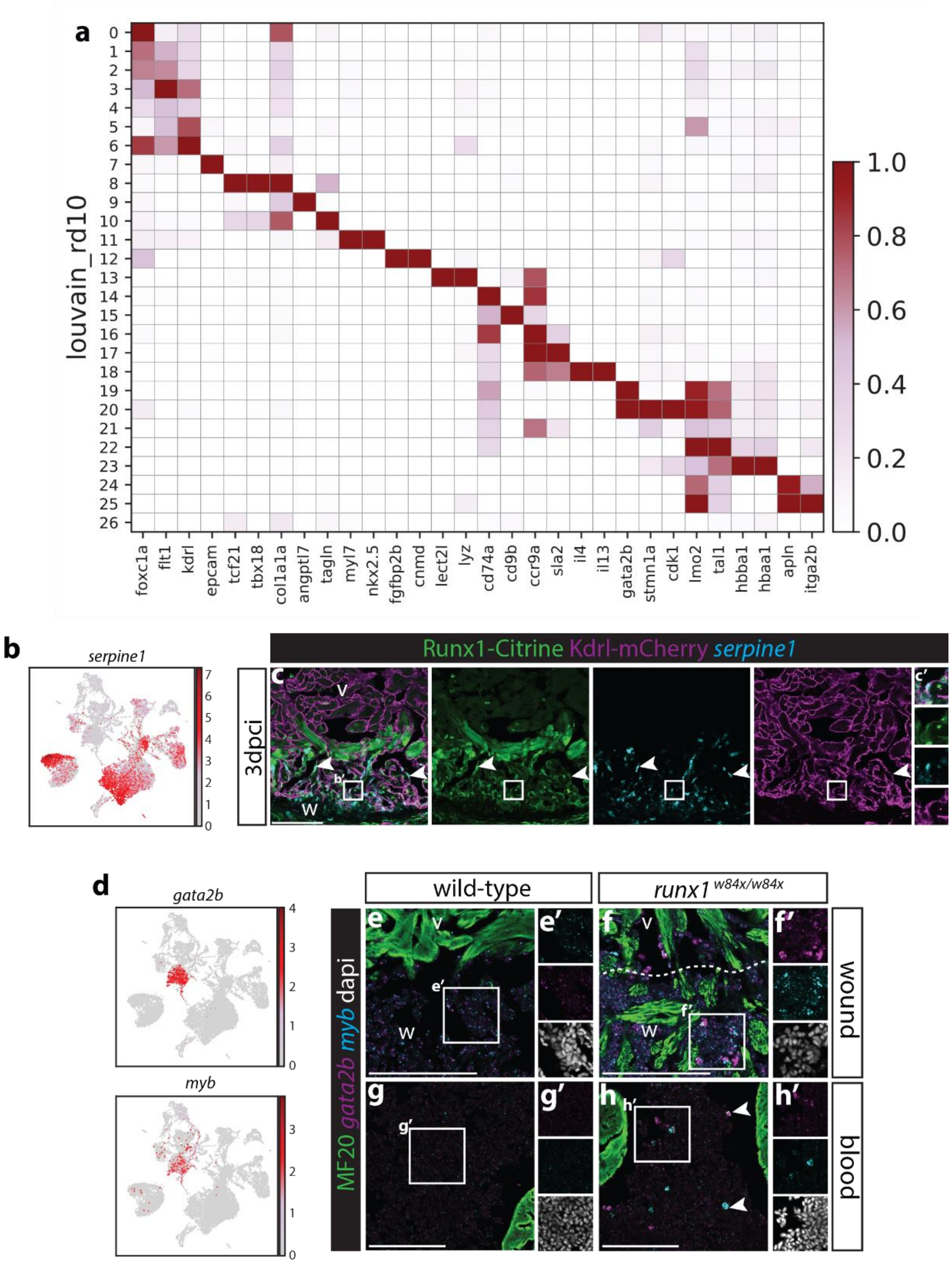

Figure S5. *Serpine1* expression overlaps with Runx1-Citrine after injury.

a, heatmap showing example genes used to determine the identity of the different cell clusters. B, UMAP plot of all cells showing expression of *serpine1*. c-c', immunohistochemistry for Citrine and mCherry combined with *in situ* hybridisation for *serpine1*. Analysis of *serpine1* on 3dpci sections shows a largely overlapping expression pattern to Runx1-Citrine in the endocardium (arrowheads and insert c'). d, UMAP plot of all cells show that *runx1* mutant specific blood cell populations have high levels of expression of *gata2b* and *myb*. e-h', immunohistochemistry for MF20 combined with *in situ* hybridisation for *gata2b* and *myb*. Inserts and arrowheads point to *runx1* mutant specific *gata2b* and *myb* expression in blood cells, both in the wound (e-f') and blood (g-h'). v, ventricle; w, wound. Scale bars depict 100 µm.

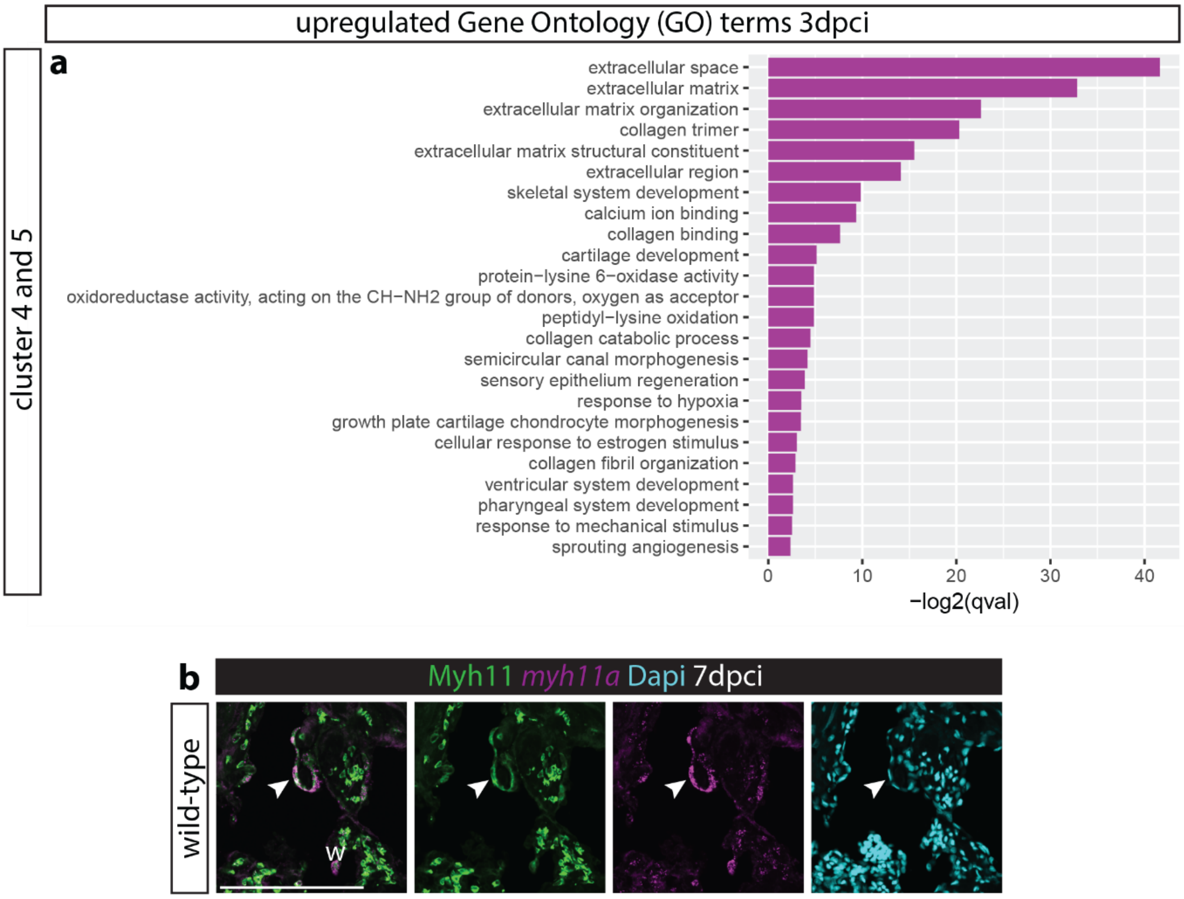

Figure S6. Upregulation of extracellular matrix genes in the *runx1-citrine* positive endocardium after injury.

a, GO term analysis shows strong upregulation of GO terms associated with extracellular matrix formation in cluster 4 and 5 of the *citrine/runx1;mcherry/kdrl* positive cells after injury. b, immunohistochemistry for Myh11 combined with *in situ* hybridisation for *myh11a* and nuclear marker Dapi. Arrowhead points to overlap of myh11a mRNA with Myh11 protein, indicating specific binding of the Myh11 antibody. w, wound. Scale bars depict 100 µm.

Development • Supplementary information
